# Supplementary figures and images for: Germination response of diverse wild and landrace chile peppers (Capsicum spp.) under drought stress simulated with polyethylene glycol
Source: PLoS One. 2020 Nov 16;15(11):e0236001. doi: 10.1371/journal.pone.0236001 (PMC7668591; doi:10.1371/journal.pone.0236001)

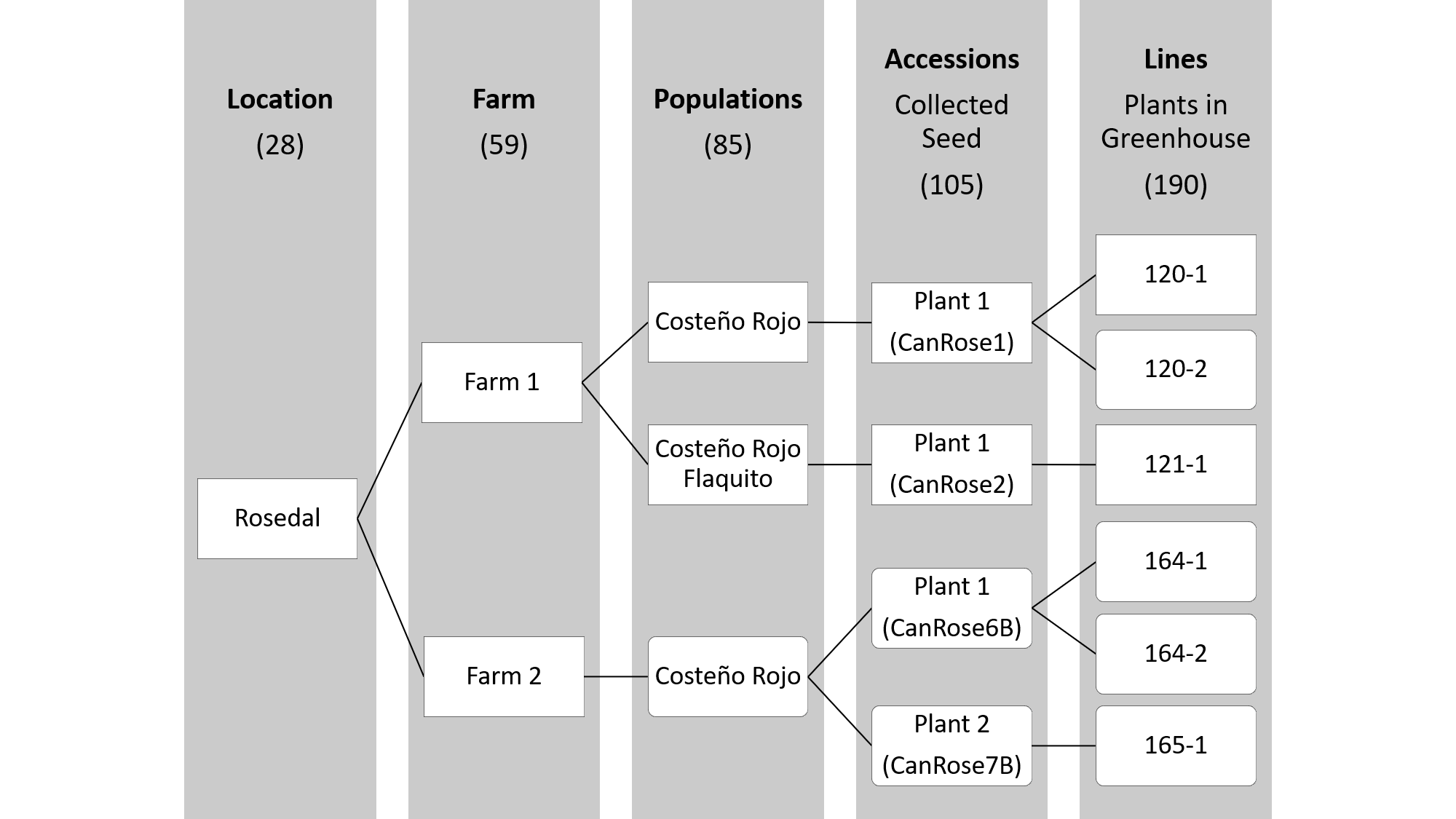

Supplement: S1 Fig — Seeds or fruits were collected from 28 different communities in Southern Mexico. In each community, several farms or locations were visited (total of 59). Seed from one or more landraces were collected at each location. A population is made up of one or more maternal plants of the same landrace from the same location. An accession represents seeds sampled from an individual plant in the field. One or two lines in the greenhouse were developed from each accession. (TIF) [file pone.0236001.s001.tif]

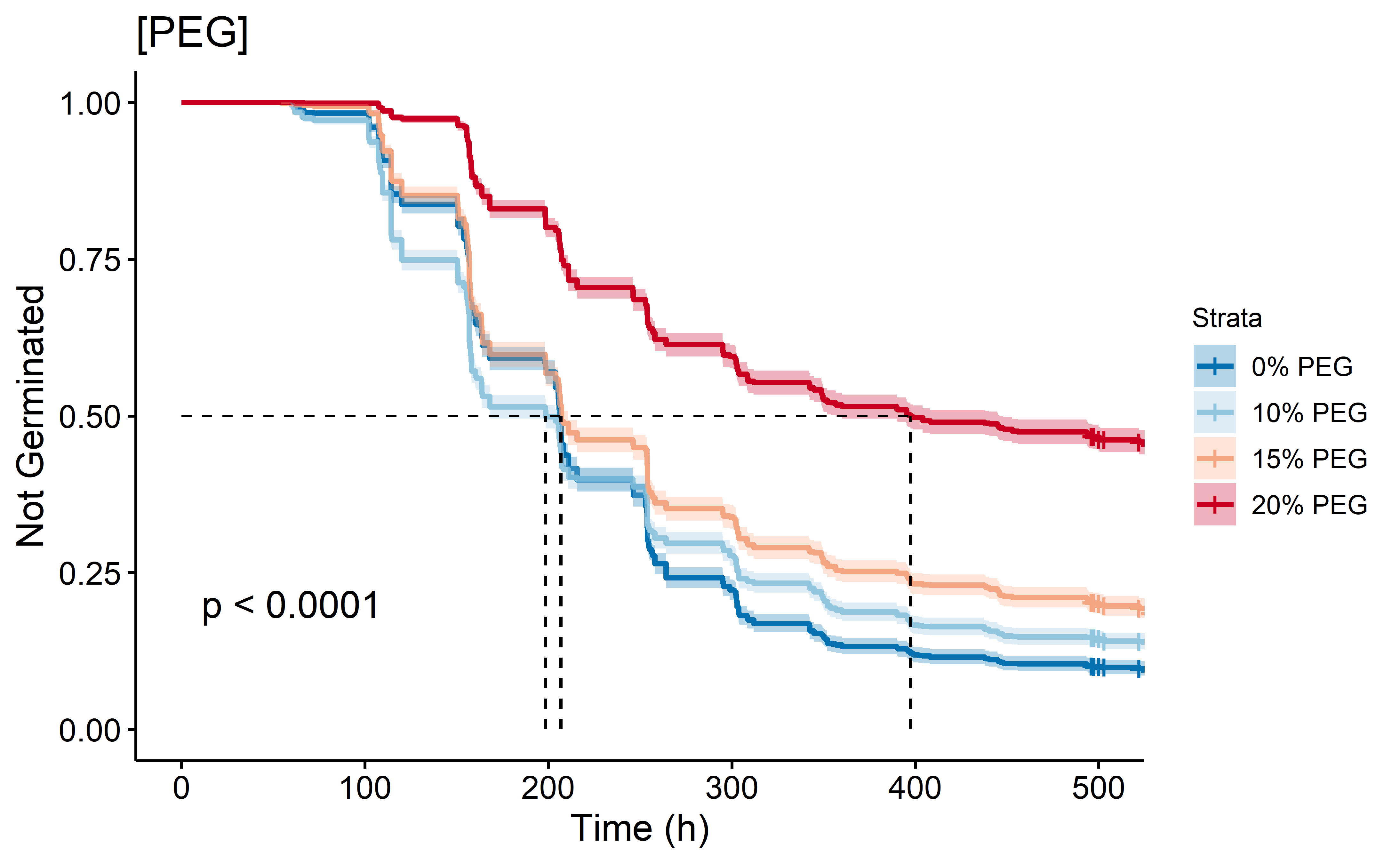

Supplement: S2 Fig — The p-value from a log-rank test compares survival distributions. The dotted line represents the time to 50% germination (t50). Pairwise comparisons of individual curves are presented in Table 1 in S3 File. (TIF) [file pone.0236001.s002.tif]

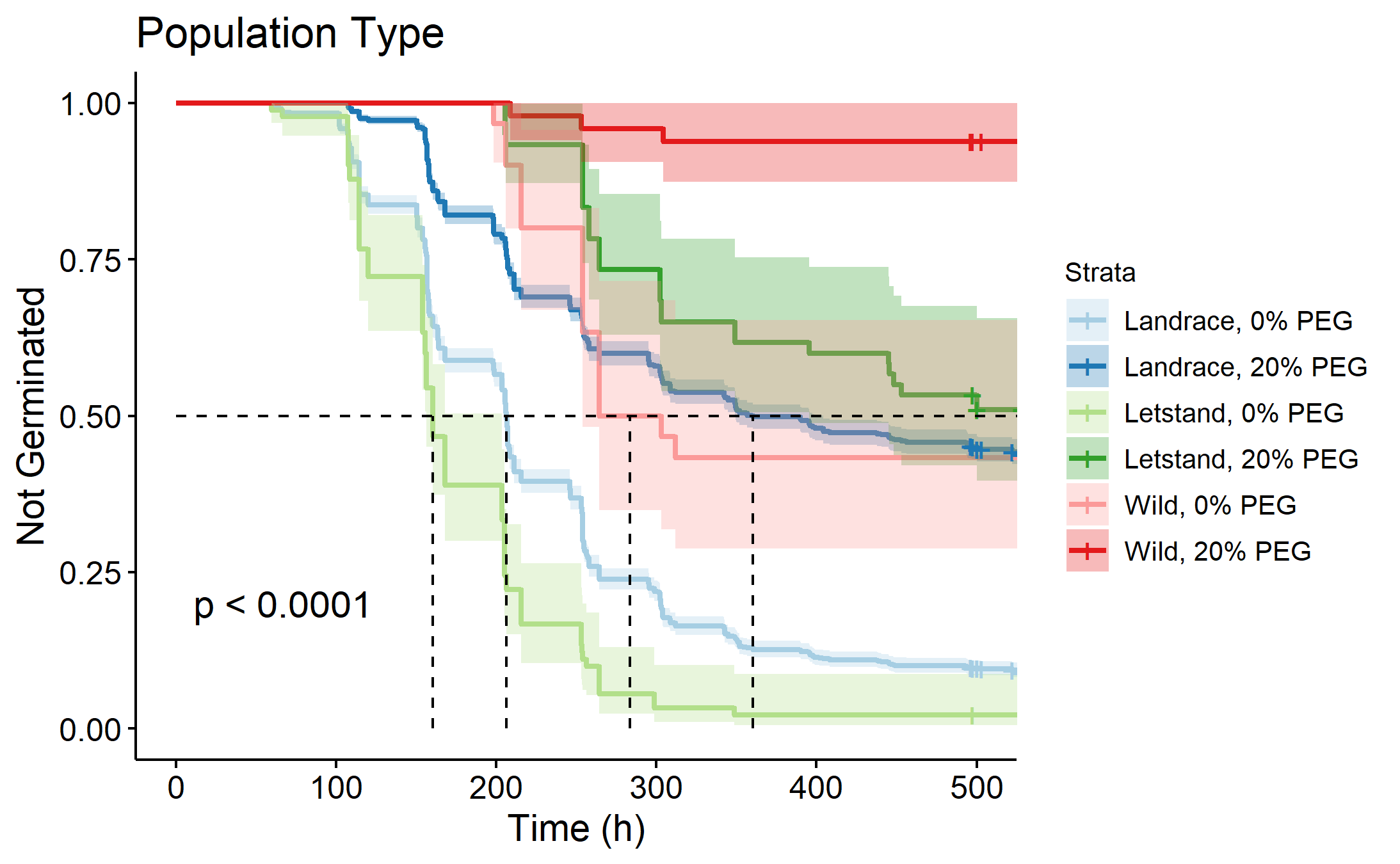

Supplement: S3 Fig — The p-value from a log-rank test compares survival distributions. The dotted line represents the time to 50% germination (t50). Pairwise comparisons of individual curves are presented in Table 4 in S3 File. (TIF) [file pone.0236001.s003.tif]
