# Supplementary material for: Germination response of diverse wild and landrace chile peppers (Capsicum spp.) under drought stress simulated with polyethylene glycol
Source: PLoS One. 2020 Nov 16;15(11):e0236001. doi: 10.1371/journal.pone.0236001 (PMC7668591; doi:10.1371/journal.pone.0236001)
Supplement: S1 Table — (PDF) [file pone.0236001.s004.pdf]

Table S1. The distribution of populations, accessions and lines across landraces studied.

| *Landrace* | *No. of Populations* | *No. of Accessions* | *No. of Lines* | *Ecozone* | *Cultivation System* | *Population Type* |
|---|---|---|---|---|---|---|
| Chigole | 1 | 2 | 3 | E Coast | backyard | landrace |
| Chile Bolita | 1 | 1 | 2 | E Coast | backyard | landrace |
| Chile de Agua | 9 | 28 | 39 | Central Valleys | milpa, plantation | landrace |
| Chile de Monte | 1 | 1 | 2 | E Coast | forest | wild |
| Costeño Amarillo | 1 | 1 | 1 | W Coast | plantation | landrace |
| Costeño Rojo | 5 | 12 | 19 | E Coast, W Coast | milpa, plantation | landrace |
| Dulce | 1 | 1 | 1 | Yucatan | milpa | landrace |
| Frutescens | 2 | 2 | 3 | E Coast | backyard | landrace, letstand |
| Guiña Dahni | 1 | 1 | 2 | E Coast | plantation | landrace |
| Mareño | 1 | 1 | 1 | E Coast | backyard | landrace |
| Mirasol | 1 | 1 | 2 | E Coast | backyard | landrace |
| Paradito | 2 | 3 | 3 | Yucatan | backyard | landrace |
| Payaso | 1 | 1 | 2 | E Coast | milpa | letstand |
| Piquín | 2 | 3 | 3 | W Coast | backyard | landrace |
| Solterito | 1 | 1 | 2 | E Coast | backyard | landrace |
| Taviche | 1 | 4 | 4 | Central Valleys | milpa | landrace |
| Tusta | 5 | 9 | 15 | E Coast, Central Valleys, Sierra Sur | backyard, milpa | landrace |
| *n = 18* | *36* | *72* | *131* | | | |
