# Supplementary material for: Germination response of diverse wild and landrace chile peppers (Capsicum spp.) under drought stress simulated with polyethylene glycol
Source: PLoS One. 2020 Nov 16;15(11):e0236001. doi: 10.1371/journal.pone.0236001 (PMC7668591; doi:10.1371/journal.pone.0236001)
Supplement: S1 File — (HTML) [file pone.0236001.s005.html]

Fig1


# Fig1

#### Vivian Bernau

#### September 25, 2020

```
library("dplyr")
```

```
## 
## Attaching package: 'dplyr'
```

```
## The following objects are masked from 'package:stats':
## 
##     filter, lag
```

```
## The following objects are masked from 'package:base':
## 
##     intersect, setdiff, setequal, union
```

```
library("readr")
library("ggplot2")
theme_set(theme_bw())
library("sf")
```

```
## Linking to GEOS 3.8.0, GDAL 3.0.4, PROJ 6.3.1
```

```
library("rnaturalearth")
library("rnaturalearthdata")
library("ggspatial")

coords <- read_csv("chile_locations.csv")
```

```
## Parsed with column specification:
## cols(
##   Region = col_character(),
##   Village = col_character(),
##   `Population type` = col_character(),
##   `Landrace name` = col_character(),
##   Cultivation = col_character(),
##   Lat1 = col_double(),
##   Long1 = col_double(),
##   Elev = col_double()
## )
```

```
latlong <- data.frame(coords[,c(1,6,7)])
unique_coords <- unique(latlong)
```

```
world <- ne_countries(scale = "medium", returnclass = "sf")
class(world)
```

```
## [1] "sf"         "data.frame"
```

```
sites <- st_as_sf(unique_coords, coords = c("Long1", "Lat1"), 
    crs = 4326, agr = "constant")

ggplot(data = world) +
    geom_sf() +
  geom_sf(data = sites, aes(fill = Region), size = 4, shape = 21) +
    coord_sf(xlim = c(-100, -86), ylim = c(12, 22), expand = T) +
    annotation_scale(location = "bl", width_hint = 0.5) + 
    annotation_north_arrow(location = "bl", which_north = "true", pad_x = unit(0.75, "in"), pad_y = unit(0.5, "in"), style = north_arrow_fancy_orienteering)
```

```
library(devtools)
```

```
## Loading required package: usethis
```

```
library(readr)
library(FactoMineR)
library(factoextra)
```

```
## Welcome! Want to learn more? See two factoextra-related books at https://goo.gl/ve3WBa
```

```
library(raster)
```

```
## Loading required package: sp
```

```
## 
## Attaching package: 'raster'
```

```
## The following object is masked from 'package:dplyr':
## 
##     select
```

```
library(sp)

#extract values from a raster file
# files <- list.files(path=("C:/Users/vmbernau/Box Sync/Bernau_chile-manuscripts/chile_germ/chile_germ_plosone/For submission/data-archive/wc2.0_30s_bio/"), pattern='.tif', full.names=TRUE)
# predictors <- stack(files)
# values <- extract(predictors, data.frame(unique_coords[,c(3,2)]))
# out<-cbind(unique_coords,values)
# write_csv(out,"bio2_data_30s.csv")

# getData('worldclim', var='bio', res=0.5, lon=-100, lat= 12, 
#         download = TRUE, path = "") #min coord
# getData('worldclim', var='bio', res=0.5, lon=-86, lat= 22, 
#         download = TRUE, path = "") #max coord
# 
# #extract values from a raster file
# files_22 <- list.files(path=("C:/Users/vmbernau/Box Sync/Bernau_chile-manuscripts/chile_germ/chile_germ_plosone/For submission/data-archive/wc0.5/"), pattern='22.bil', full.names=TRUE)
# files_23 <- list.files(path=("C:/Users/vmbernau/Box Sync/Bernau_chile-manuscripts/chile_germ/chile_germ_plosone/For submission/data-archive/wc0.5/"), pattern='23.bil', full.names=TRUE)
# predictors_22 <- stack(files_22)
# predictors_23 <- stack(files_23)
# predictors <- raster::merge(predictors_22, predictors_23, overlap = F)
# values <- extract(predictors, data.frame(unique_coords[,c(3,2)]))
# out<-cbind(unique_coords,values)
# write_csv(out,"bio_data_0-5.csv")

#bio_data <- read_csv("bio_data_0-5.csv")
bio_data <- read_csv("bio2_data_30s.csv")
```

```
## Parsed with column specification:
## cols(
##   .default = col_double(),
##   Region = col_character()
## )
```

```
## See spec(...) for full column specifications.
```

```
bio.pca <- PCA(bio_data[,c(4:22)], scale.unit = T, ncp = 4)
```

```
## Warning in PCA(bio_data[, c(4:22)], scale.unit = T, ncp = 4): Missing
## values are imputed by the mean of the variable: you should use the
## imputePCA function of the missMDA package
```

```
fviz_pca_ind(bio.pca, pointsize = 3, label = "none", pointshape = 19, 
             col.ind = bio_data$Region, addEllipses = T, ellipse.level = 0.95) + 
  labs(title = NULL)
```

```
## Too few points to calculate an ellipse
## Too few points to calculate an ellipse
```

```
sessionInfo()
```

```
## R version 3.5.3 (2019-03-11)
## Platform: x86_64-w64-mingw32/x64 (64-bit)
## Running under: Windows 10 x64 (build 19041)
## 
## Matrix products: default
## 
## locale:
## [1] LC_COLLATE=English_United States.1252 
## [2] LC_CTYPE=English_United States.1252   
## [3] LC_MONETARY=English_United States.1252
## [4] LC_NUMERIC=C                          
## [5] LC_TIME=English_United States.1252    
## 
## attached base packages:
## [1] stats     graphics  grDevices utils     datasets  methods   base     
## 
## other attached packages:
##  [1] raster_2.9-23           sp_1.3-1               
##  [3] factoextra_1.0.7        FactoMineR_1.42        
##  [5] devtools_2.1.0          usethis_1.5.1          
##  [7] ggspatial_1.1.4         rnaturalearthdata_0.1.0
##  [9] rnaturalearth_0.1.0     sf_0.9-6               
## [11] ggplot2_3.3.2           readr_1.3.1            
## [13] dplyr_0.8.3            
## 
## loaded via a namespace (and not attached):
##  [1] ggrepel_0.8.2        Rcpp_1.0.2           lattice_0.20-38     
##  [4] prettyunits_1.0.2    class_7.3-15         ps_1.3.0            
##  [7] assertthat_0.2.1     rprojroot_1.3-2      digest_0.6.20       
## [10] R6_2.4.0             backports_1.1.4      evaluate_0.14       
## [13] e1071_1.7-2          pillar_1.4.2         rlang_0.4.7         
## [16] callr_3.3.1          rmarkdown_1.14       labeling_0.3        
## [19] desc_1.2.0           stringr_1.4.0        munsell_0.5.0       
## [22] compiler_3.5.3       xfun_0.8             pkgconfig_2.0.2     
## [25] pkgbuild_1.0.3       rgeos_0.4-3          htmltools_0.5.0     
## [28] flashClust_1.01-2    tidyselect_1.1.0     tibble_2.1.3        
## [31] codetools_0.2-16     ggpubr_0.2.2         crayon_1.3.4        
## [34] withr_2.1.2          MASS_7.3-51.1        leaps_3.0           
## [37] grid_3.5.3           gtable_0.3.0         DBI_1.0.0           
## [40] magrittr_1.5         units_0.6-3          scales_1.0.0        
## [43] KernSmooth_2.23-15   cli_1.1.0            stringi_1.4.3       
## [46] ggsignif_0.6.0       fs_1.3.1             remotes_2.1.0       
## [49] scatterplot3d_0.3-41 testthat_2.2.0       vctrs_0.3.4         
## [52] tools_3.5.3          glue_1.4.2           purrr_0.3.2         
## [55] hms_0.5.0            processx_3.4.1       pkgload_1.0.2       
## [58] yaml_2.2.0           colorspace_1.4-1     cluster_2.0.7-1     
## [61] sessioninfo_1.1.1    classInt_0.4-3       memoise_1.1.0       
## [64] knitr_1.23
```
