# Supplementary material for: Germination response of diverse wild and landrace chile peppers (Capsicum spp.) under drought stress simulated with polyethylene glycol
Source: PLoS One. 2020 Nov 16;15(11):e0236001. doi: 10.1371/journal.pone.0236001 (PMC7668591; doi:10.1371/journal.pone.0236001)
Supplement: S2 File — (HTML) [file pone.0236001.s006.html]

SURVIVAL


# SURVIVAL

#### Vivian Bernau

#### 30 January 2018

6 Sept 2017

Based on McNair et al 2012; Seed Science Research

### NONPARAMETRIC ANALYSIS

1. Characterizing pattern of germination within groups –> Kaplan-Meier test: survfit()
2. Comparing patterns of germination in groups –> Fleming-Harrington test(?): survdiff()

My data is right-sensored. My data is interval, but can probably be analyzed nonparametrically as exact for as long as plates with large losses are removed.

# Survivor function: probability that the germination time is greater than t

```
library(pacman)
p_load(survival, mnormt, survminer, RColorBrewer, knitr, kableExtra, dplyr, 
    DT, ggpubr)
```

```
# read in germination data in pre-lifetab format
df <- read.csv(paste(path, "/germ_data.csv", sep = ""), header = T, na.strings = c("", 
    "NA"))
str(df)
```

```
## 'data.frame':    10773 obs. of  22 variables:
##  $ sampleid       : Factor w/ 76 levels "CanAbasolo1",..: 1 1 1 1 1 1 1 1 1 1 ...
##  $ line           : Factor w/ 120 levels "100-1","100-2",..: 96 96 96 96 96 96 96 96 96 96 ...
##  $ X              : num  915 913 3081 914 917 ...
##  $ rep            : int  1 1 2 1 1 2 2 2 1 2 ...
##  $ run            : int  1 1 3 1 1 3 3 3 1 3 ...
##  $ shelf          : int  7 7 5 7 7 5 5 3 7 5 ...
##  $ plate          : int  237 237 170 237 237 170 170 78 237 170 ...
##  $ trt            : int  10 10 10 10 10 10 10 20 10 10 ...
##  $ end            : num  246 102 158 151 542 ...
##  $ status         : int  1 1 1 1 0 1 1 0 0 0 ...
##  $ viable         : int  1 1 1 1 1 1 1 0 0 0 ...
##  $ number         : int  1 4 6 1 2 2 2 2 1 1 ...
##  $ pedigree       : Factor w/ 119 levels "14CAg128-1","14CAg128-2",..: 45 45 45 45 45 45 45 45 45 45 ...
##  $ planting.date  : Factor w/ 1 level "11/10/2014": 1 1 1 1 1 1 1 1 1 1 ...
##  $ landrace.abb   : Factor w/ 22 levels "CAg","Cam","CdA",..: 1 1 1 1 1 1 1 1 1 1 ...
##  $ region         : Factor w/ 5 levels "central-valleys",..: 1 1 1 1 1 1 1 1 1 1 ...
##  $ pop            : Factor w/ 36 levels "CAg_24","CAg_25",..: 9 9 9 9 9 9 9 9 9 9 ...
##  $ population.type: Factor w/ 3 levels "landrace","letstand",..: 1 1 1 1 1 1 1 1 1 1 ...
##  $ landrace       : Factor w/ 17 levels "Chigole","Chile Bolita",..: 3 3 3 3 3 3 3 3 3 3 ...
##  $ cultivation    : Factor w/ 4 levels "Backyard","Forest",..: 3 3 3 3 3 3 3 3 3 3 ...
##  $ main.use       : Factor w/ 3 levels "Dry","Fresh",..: NA NA NA NA NA NA NA NA NA NA ...
##  $ uniqueplate    : Factor w/ 1084 levels "1_1","1_10","1_100",..: 84 84 423 84 84 423 423 524 84 423 ...
```

```
df$end <- round(df$end, digits = 1)
df$run <- as.factor(df$run)

df <- subset(df, region != "control")
df$cv <- as.numeric(df$region == "central valleys")
df$ecoast <- as.numeric(df$region == "ecoast")
df$wcoast <- as.numeric(df$region == "wcoast")
df$yucatan <- as.numeric(df$region == "yucatan")
df$ss <- as.numeric(df$region == "sierra madre")
```

```
test.peg <- survfit(Surv(end, status) ~ trt, data = df, type = "kaplan-meier", 
    conf.type = "log-log")
labels.peg <- c("0% PEG", "10% PEG", "15% PEG", "20% PEG")
ggsurvplot(test.peg, data = df, conf.int = T, pval = T, title = "[PEG]", surv.median.line = "hv", 
    xlab = "Time (h)", ylab = "Not Germinated", legend = "right", legend.labs = labels.peg, 
    palette = rev(brewer.pal(4, "RdBu"))) + guides(col = guide_legend(ncol = 1))
```

```
# ggsave('peg.jpg', dpi = 600) ggsave('FigS1.tiff', dpi = 600)
survdiff.peg <- pairwise_survdiff(Surv(end, status) ~ trt, data = df, p.adjust.method = "bonferroni", 
    rho = 0)
survdiff.peg$p.value %>% kable() %>% kable_styling()
```

|  | 0 | 10 | 15 |
| --- | --- | --- | --- |
| 10 | 0.4846447 | NA | NA |
| 15 | 0.0000000 | 0 | NA |
| 20 | 0.0000000 | 0 | 0 |

```
# sink('peg.txt'); print(survdiff.peg$p.value); sink()
```

```
# fit and plot Kaplan-Meier survivor function, includes 95% confidence
# intervals
df_0_20 <- subset(df, trt %in% c(0, 20))
labels <- c("Central Valleys, 0% PEG", "Central Valleys, 20% PEG", "E Coast, 0% PEG", 
    "E Coast, 20% PEG", "Sierra Sur, 0% PEG", "Sierra Sur, 20% PEG", "W Coast, 0% PEG", 
    "W Coast, 20% PEG", "Yucatan, 0% PEG", "Yucatan, 20% PEG")
test.pegregion.0.20 <- survfit(Surv(end, status) ~ region + trt, data = df_0_20, 
    type = "kaplan-meier", conf.type = "log-log")
ggsurvplot(test.pegregion.0.20, data = df_0_20, conf.int = T, pval = T, surv.median.line = "hv", 
    xlab = "Time (h)", ylab = "Not Germinated", legend = "right", palette = "Paired", 
    legend.labs = paste(labels)) + guides(col = guide_legend(ncol = 1))
```

```
# ggsave('peg_region_0-20.jpg', dpi = 600) ggsave('Fig2.tiff', dpi = 600)
survdiff.pegregion.0.20 <- pairwise_survdiff(Surv(end, status) ~ trt + region, 
    data = df_0_20, p.adjust.method = "bonferroni", rho = 0)
survdiff.pegregion.0.20$p.value %>% kable() %>% kable_styling()
```

|  | trt=0, region=central-valleys | trt=0, region=ecoast | trt=0, region=sierra-sur | trt=0, region=wcoast | trt=0, region=yucatan | trt=20, region=central-valleys | trt=20, region=ecoast | trt=20, region=sierra-sur | trt=20, region=wcoast |
| --- | --- | --- | --- | --- | --- | --- | --- | --- | --- |
| trt=0, region=ecoast | 0.0006912 | NA | NA | NA | NA | NA | NA | NA | NA |
| trt=0, region=sierra-sur | 1.0000000 | 1.0000000 | NA | NA | NA | NA | NA | NA | NA |
| trt=0, region=wcoast | 0.0025166 | 0.0000000 | 0.003319 | NA | NA | NA | NA | NA | NA |
| trt=0, region=yucatan | 1.0000000 | 0.1450157 | 1.000000 | 0.0096082 | NA | NA | NA | NA | NA |
| trt=20, region=central-valleys | 0.0000000 | 0.0000000 | 0.000000 | 0.0000000 | 0.0000000 | NA | NA | NA | NA |
| trt=20, region=ecoast | 0.0000000 | 0.0000000 | 0.000000 | 0.0000000 | 0.0000000 | 0 | NA | NA | NA |
| trt=20, region=sierra-sur | 0.0000000 | 0.0000000 | 0.000000 | 0.0000000 | 0.0000000 | 1 | 9e-07 | NA | NA |
| trt=20, region=wcoast | 0.0023263 | 1.0000000 | 1.000000 | 0.0000000 | 0.7961364 | 0 | 0e+00 | 7.47e-05 | NA |
| trt=20, region=yucatan | 0.0000000 | 0.0000000 | 0.000000 | 0.0000000 | 0.0000000 | 0 | 1e+00 | 2.58e-05 | 0 |

```
# sink('peg_region_0-20.txt'); print(survdiff.pegregion.0.20$p.value);
# sink()
```

```
# TESTING DIFFERENCES ACROSS CULTIVATION SYSTEMS
df_cult <- subset(df_0_20, cultivation %in% c("Backyard", "Forest", "Milpa", 
    "Plantation"))
labels.cult <- c("Backyard, 0% PEG", "Backyard, 20% PEG", "Forest, 0% PEG", 
    "Forest, 20% PEG", "Milpa, 0% PEG", "Milpa, 20% PEG", "Plantation, 0% PEG", 
    "Plantation, 20% PEG")
test.cult <- survfit(Surv(end, status) ~ cultivation + trt, data = df_cult, 
    type = "kaplan-meier", na.action = na.omit)
ggsurvplot(test.cult, data = df_0_20, pval = T, title = "Cultivation Systems x [PEG]", 
    surv.median.line = "hv", palette = "Paired", conf.int = T, legend = "right", 
    xlab = "Time (h)", ylab = "Not Germinated", legend.labs = labels.cult) + 
    guides(col = guide_legend(ncol = 1))
```

```
# ggsave('peg_cult_0-20.jpg', dpi = 600) ggsave('Fig3.tiff', dpi = 600)

survdiff_cult <- pairwise_survdiff(Surv(end, status) ~ cultivation + trt, data = df_cult, 
    p.adjust.method = "bonferroni", rho = 0)
survdiff_cult$p.value %>% kable() %>% kable_styling()
```

|  | cultivation=Backyard, trt=0 | cultivation=Backyard, trt=20 | cultivation=Forest, trt=0 | cultivation=Forest, trt=20 | cultivation=Milpa, trt=0 | cultivation=Milpa, trt=20 | cultivation=Plantation, trt=0 |
| --- | --- | --- | --- | --- | --- | --- | --- |
| cultivation=Backyard, trt=20 | 0.0000000 | NA | NA | NA | NA | NA | NA |
| cultivation=Forest, trt=0 | 0.0001541 | 0.0084102 | NA | NA | NA | NA | NA |
| cultivation=Forest, trt=20 | 0.0000000 | 0.0265231 | 0.0000071 | NA | NA | NA | NA |
| cultivation=Milpa, trt=0 | 0.0000000 | 0.0000000 | 0.0000010 | 0 | NA | NA | NA |
| cultivation=Milpa, trt=20 | 0.0000000 | 0.0000000 | 1.0000000 | 0 | 0 | NA | NA |
| cultivation=Plantation, trt=0 | 0.0000000 | 0.0000000 | 0.0000184 | 0 | 1 | 0.0000000 | NA |
| cultivation=Plantation, trt=20 | 0.0000000 | 0.0000000 | 0.6657088 | 0 | 0 | 0.9688345 | 0 |

```
# sink('peg_cult_0-20.txt'); print(survdiff_cult$p.value); sink()
```

```
labels.domestication <- c("Landrace, 0% PEG", "Landrace, 20% PEG", "Letstand, 0% PEG", 
    "Letstand, 20% PEG", "Wild, 0% PEG", "Wild, 20% PEG")
test.domestication <- survfit(Surv(end, status) ~ population.type + trt, data = df_0_20, 
    type = "kaplan-meier", na.action = na.omit)
ggsurvplot(test.domestication, data = df_0_20, pval = T, conf.int = T, legend = "right", 
    xlab = "Time (h)", palette = "Paired", surv.median.line = "hv", ylab = "Not Germinated", 
    title = "Population type x [PEG]", legend.labs = labels.domestication)
```

```
# ggsave('peg_dom_0-20.jpg', units = 'in', dpi = 600) ggsave('FigS3.tiff',
# units = 'in', dpi = 600)
survdiff_domestication <- pairwise_survdiff(Surv(end, status) ~ population.type + 
    trt, data = df_0_20, p.adjust.method = "bonferroni", rho = 0)

survdiff_domestication$p.value %>% kable() %>% kable_styling
```

|  | population.type=landrace, trt=0 | population.type=landrace, trt=20 | population.type=letstand, trt=0 | population.type=letstand, trt=20 | population.type=wild, trt=0 |
| --- | --- | --- | --- | --- | --- |
| population.type=landrace, trt=20 | 0.00e+00 | NA | NA | NA | NA |
| population.type=letstand, trt=0 | 6.00e-07 | 0e+00 | NA | NA | NA |
| population.type=letstand, trt=20 | 0.00e+00 | 1e+00 | 0 | NA | NA |
| population.type=wild, trt=0 | 3.02e-05 | 1e+00 | 0 | 1.00e+00 | NA |
| population.type=wild, trt=20 | 0.00e+00 | 2e-07 | 0 | 4.23e-05 | 3.8e-06 |

```
# sink('peg_dom_0-20.txt'); print(survdiff_domestication$p.value); sink()
```

```
# TESTING DIFFERENT LANDRACES
summary(df_0_20$landrace)
```

```
##          Chigole     Chile Bolita    Chile de Agua   Chile de Monte 
##              199              621             1031               79 
## Costeno Amarillo     Costeno Rojo            Dulce       Frutescens 
##               70             1103               69              188 
##      Guina Dahni           Mareno          Mirasol         Paradito 
##              147               69              110              308 
##           Payaso           Piquin        Solterito          Taviche 
##               90              130              108              130 
##            Tusta             NA's 
##              697               70
```

```
labels.ecoast <- c("Chigole, 0% PEG", "Chigole, 20% PEG", "Chile Bolita, 0% PEG", 
    "Chile Bolita, 20% PEG", "Chile de Monte, 0% PEG", "Chile de Monte, 20% PEG", 
    "Costeno Rojo, 0% PEG", "Costeno Rojo, 20% PEG", "Frutescens, 0% PEG", "Frutescens, 20% PEG", 
    "Guina Dahni, 0% PEG", "Guina Dahni, 20% PEG", "Mareno, 0% PEG", "Mareno, 20% PEG", 
    "Mirasol, 0% PEG", "Mirasol, 20% PEG", "Payaso, 0% PEG", "Payaso, 20% PEG", 
    "Solterito, 0% PEG", "Solterito, 20% PEG", "Tusta, 0% PEG", "Tusta, 20% PEG")
linetype.ecoast <- c(1, 3, 1, 3, 1, 3, 1, 3, 1, 3, 1, 3, 1, 3, 1, 3, 1, 3, 1, 
    3, 1, 3)
df_ecoast <- subset(df_0_20, region == "ecoast")
test.landrace.ecoast <- survfit(Surv(end, status) ~ landrace + trt, data = df_ecoast, 
    type = "kaplan-meier", conf.type = "log-log", na.action = na.exclude)

ggsurvplot(test.landrace.ecoast, data = df_0_20, conf.int = T, pval = T, xlab = "Time (h)", 
    legend = "right", ylab = "Not Germinated", surv.median.line = "hv", title = "E Coast", 
    legend.labs = labels.ecoast, linetype = linetype.ecoast)
```

```
# ggsave(file = 'ecoast_landrace.jpg', dpi = 600, width = 10, units = 'in')
# ggsave(file = 'ecoast_landrace.tiff', dpi = 600, width = 10, units = 'in')
survdiff_lr_ecoast <- pairwise_survdiff(Surv(end, status) ~ landrace + trt, 
    data = df_ecoast, p.adjust.method = "bonferroni", rho = 0)
survdiff_lr_ecoast$p.value %>% kable() %>% kable_styling
```

|  | landrace=Chigole, trt=0 | landrace=Chigole, trt=20 | landrace=Chile Bolita, trt=0 | landrace=Chile Bolita, trt=20 | landrace=Chile de Monte, trt=0 | landrace=Chile de Monte, trt=20 | landrace=Costeno Rojo, trt=0 | landrace=Costeno Rojo, trt=20 | landrace=Frutescens, trt=0 | landrace=Frutescens, trt=20 | landrace=Guina Dahni, trt=0 | landrace=Guina Dahni, trt=20 | landrace=Mareno, trt=0 | landrace=Mareno, trt=20 | landrace=Mirasol, trt=0 | landrace=Mirasol, trt=20 | landrace=Payaso, trt=0 | landrace=Payaso, trt=20 | landrace=Solterito, trt=0 | landrace=Solterito, trt=20 | landrace=Tusta, trt=0 |
| --- | --- | --- | --- | --- | --- | --- | --- | --- | --- | --- | --- | --- | --- | --- | --- | --- | --- | --- | --- | --- | --- |
| landrace=Chigole, trt=20 | 0.0000000 | NA | NA | NA | NA | NA | NA | NA | NA | NA | NA | NA | NA | NA | NA | NA | NA | NA | NA | NA | NA |
| landrace=Chile Bolita, trt=0 | 1.0000000 | 0.0000000 | NA | NA | NA | NA | NA | NA | NA | NA | NA | NA | NA | NA | NA | NA | NA | NA | NA | NA | NA |
| landrace=Chile Bolita, trt=20 | 0.0000000 | 1.0000000 | 0.0000000 | NA | NA | NA | NA | NA | NA | NA | NA | NA | NA | NA | NA | NA | NA | NA | NA | NA | NA |
| landrace=Chile de Monte, trt=0 | 0.0027673 | 1.0000000 | 0.0020033 | 0.4331550 | NA | NA | NA | NA | NA | NA | NA | NA | NA | NA | NA | NA | NA | NA | NA | NA | NA |
| landrace=Chile de Monte, trt=20 | 0.0000000 | 0.0065644 | 0.0000000 | 0.1301815 | 0.0000584 | NA | NA | NA | NA | NA | NA | NA | NA | NA | NA | NA | NA | NA | NA | NA | NA |
| landrace=Costeno Rojo, trt=0 | 1.0000000 | 0.0000000 | 1.0000000 | 0.0000000 | 0.0057777 | 0.0000000 | NA | NA | NA | NA | NA | NA | NA | NA | NA | NA | NA | NA | NA | NA | NA |
| landrace=Costeno Rojo, trt=20 | 0.0008115 | 0.0223847 | 0.0000040 | 0.0000000 | 1.0000000 | 0.0000001 | 0.0000512 | NA | NA | NA | NA | NA | NA | NA | NA | NA | NA | NA | NA | NA | NA |
| landrace=Frutescens, trt=0 | 1.0000000 | 0.0000000 | 1.0000000 | 0.0000000 | 0.1277898 | 0.0000000 | 1.0000000 | 1.0000000 | NA | NA | NA | NA | NA | NA | NA | NA | NA | NA | NA | NA | NA |
| landrace=Frutescens, trt=20 | 0.0000000 | 0.0650117 | 0.0000000 | 1.0000000 | 0.0015073 | 1.0000000 | 0.0000000 | 0.0000000 | 0.0000000 | NA | NA | NA | NA | NA | NA | NA | NA | NA | NA | NA | NA |
| landrace=Guina Dahni, trt=0 | 0.0000000 | 0.0000000 | 0.0000000 | 0.0000000 | 0.0000000 | 0.0000000 | 0.0000000 | 0.0000000 | 0.0000000 | 0.0000000 | NA | NA | NA | NA | NA | NA | NA | NA | NA | NA | NA |
| landrace=Guina Dahni, trt=20 | 0.0000086 | 1.0000000 | 0.0000021 | 0.0000099 | 1.0000000 | 0.0000002 | 0.0000073 | 1.0000000 | 0.0213976 | 0.0000000 | 0.0000000 | NA | NA | NA | NA | NA | NA | NA | NA | NA | NA |
| landrace=Mareno, trt=0 | 1.0000000 | 0.0000000 | 1.0000000 | 0.0000000 | 0.0009129 | 0.0000000 | 1.0000000 | 0.0108672 | 0.2883656 | 0.0000000 | 0.0000749 | 0.0000016 | NA | NA | NA | NA | NA | NA | NA | NA | NA |
| landrace=Mareno, trt=20 | 0.0000000 | 1.0000000 | 0.0000000 | 1.0000000 | 0.1015723 | 1.0000000 | 0.0000000 | 0.0037210 | 0.0000000 | 1.0000000 | 0.0000000 | 0.0054622 | 0.0000000 | NA | NA | NA | NA | NA | NA | NA | NA |
| landrace=Mirasol, trt=0 | 0.1598541 | 0.0000093 | 0.3113886 | 0.0000000 | 1.0000000 | 0.0000000 | 0.1101969 | 1.0000000 | 1.0000000 | 0.0000000 | 0.0000000 | 1.0000000 | 0.0036549 | 0.0000000 | NA | NA | NA | NA | NA | NA | NA |
| landrace=Mirasol, trt=20 | 0.0000000 | 0.4325206 | 0.0000000 | 1.0000000 | 0.0071306 | 1.0000000 | 0.0000000 | 0.0000441 | 0.0000000 | 1.0000000 | 0.0000000 | 0.0001270 | 0.0000000 | 1.0000000 | 0.0000000 | NA | NA | NA | NA | NA | NA |
| landrace=Payaso, trt=0 | 0.0000002 | 0.0000000 | 0.0000000 | 0.0000000 | 0.0000000 | 0.0000000 | 0.0000015 | 0.0000000 | 0.0000000 | 0.0000000 | 0.8167693 | 0.0000000 | 0.3549242 | 0.0000000 | 0.0000000 | 0.000000 | NA | NA | NA | NA | NA |
| landrace=Payaso, trt=20 | 0.4409463 | 1.0000000 | 0.5460932 | 0.0151737 | 1.0000000 | 0.0000007 | 0.5988927 | 1.0000000 | 1.0000000 | 0.0000136 | 0.0000000 | 1.0000000 | 0.1123283 | 0.0026384 | 1.0000000 | 0.000124 | 9e-07 | NA | NA | NA | NA |
| landrace=Solterito, trt=0 | 0.0969637 | 0.0340006 | 0.0699349 | 0.0000000 | 1.0000000 | 0.0000000 | 0.1490743 | 1.0000000 | 1.0000000 | 0.0000000 | 0.0000000 | 1.0000000 | 0.0469904 | 0.0000963 | 1.0000000 | 0.000001 | 0e+00 | 1.0000000 | NA | NA | NA |
| landrace=Solterito, trt=20 | 0.0000000 | 0.0050512 | 0.0000000 | 0.1600808 | 0.0000553 | 1.0000000 | 0.0000000 | 0.0000000 | 0.0000000 | 1.0000000 | 0.0000000 | 0.0000000 | 0.0000000 | 1.0000000 | 0.0000000 | 1.000000 | 0e+00 | 0.0000001 | 0.0000000 | NA | NA |
| landrace=Tusta, trt=0 | 1.0000000 | 0.0000000 | 1.0000000 | 0.0000000 | 0.0030697 | 0.0000000 | 1.0000000 | 0.0018997 | 1.0000000 | 0.0000000 | 0.0000000 | 0.0000024 | 1.0000000 | 0.0000000 | 0.0467642 | 0.000000 | 0e+00 | 0.3976805 | 0.0603216 | 0.0000000 | NA |
| landrace=Tusta, trt=20 | 0.0000000 | 1.0000000 | 0.0000000 | 1.0000000 | 1.0000000 | 0.0327162 | 0.0000000 | 0.0000008 | 0.0000000 | 0.3797195 | 0.0000000 | 0.0180899 | 0.0000000 | 1.0000000 | 0.0000000 | 1.000000 | 0e+00 | 0.7235725 | 0.0000190 | 0.0224451 | 0 |

```
# sink('ecoast_landrace.txt'); print(survdiff_lr_ecoast$p.value); sink()

labels.ecoast.1 <- c("Chigole, 0% PEG", "Chile Bolita, 0% PEG", "Chile de Monte, 0% PEG", 
    "Costeno Rojo, 0% PEG", "Frutescens, 0% PEG", "Guina Dahni, 0% PEG", "Mareno, 0% PEG", 
    "Mirasol, 0% PEG", "Payaso, 0% PEG", "Solterito, 0% PEG", "Tusta, 0% PEG")
df_ecoast_1 <- subset(df_ecoast, trt == 0)
test.landrace.ecoast.1 <- survfit(Surv(end, status) ~ landrace + trt, data = df_ecoast_1, 
    type = "kaplan-meier", conf.type = "log-log", na.action = na.exclude)

ggsurvplot(test.landrace.ecoast.1, data = df_ecoast_1, conf.int = T, pval = T, 
    xlab = NULL, legend = "right", ylab = "Not Germinated", surv.median.line = "hv", 
    title = "A) E Coast, 0% PEG", legend.labs = labels.ecoast.1)
```

```
# ggsave(file = 'ecoast_landrace_0.jpg', dpi = 600)
ggsave(file = "Fig4E.tiff", dpi = 600)
```

```
## Saving 7 x 5 in image
```

```
labels.ecoast.2 <- c("Chigole, 20% PEG", "Chile Bolita, 20% PEG", "Chile de Monte, 20% PEG", 
    "Costeno Rojo, 20% PEG", "Frutescens, 20% PEG", "Guina Dahni, 20% PEG", 
    "Mareno, 20% PEG", "Mirasol, 20% PEG", "Payaso, 20% PEG", "Solterito, 20% PEG", 
    "Tusta, 20% PEG")
df_ecoast_2 <- subset(df_ecoast, trt == 20)
test.landrace.ecoast.2 <- survfit(Surv(end, status) ~ landrace + trt, data = df_ecoast_2, 
    type = "kaplan-meier", conf.type = "log-log", na.action = na.exclude)
ggsurvplot(test.landrace.ecoast.2, data = df_ecoast_2, conf.int = T, pval = T, 
    xlab = "Time (h)", legend = "right", ylab = "Not Germinated", surv.median.line = "hv", 
    title = "B) E Coast, 20% PEG", legend.labs = labels.ecoast.2)
```

```
# ggsave(file = 'ecoast_landrace_20.jpg', dpi = 600)
ggsave(file = "Fig4F.tiff", dpi = 600)
```

```
## Saving 7 x 5 in image
```

```
labels.cv <- c("Chile de Agua, 0% PEG", "Chile de Agua, 20% PEG", "Taviche, 0% PEG", 
    "Taviche, 20% PEG", "Tusta, 0% PEG", "Tusta, 20% PEG")
df_cv <- subset(df_0_20, region == "central-valleys")
test.landrace.cv <- survfit(Surv(end, status) ~ landrace + trt, data = df_cv, 
    type = "kaplan-meier", conf.type = "log-log", na.action = na.exclude)
ggsurvplot(test.landrace.cv, data = df_cv, conf.int = T, pval = T, xlab = "Time (h)", 
    legend = "right", ylab = "Not Germinated", surv.median.line = "hv", palette = "Paired", 
    title = "C) Central Valleys", legend.labs = labels.cv)
```

```
# ggsave(file = 'central-valleys_landrace.jpg', dpi = 600)
ggsave(file = "Fig4C.tiff", dpi = 600)
```

```
## Saving 7 x 5 in image
```

```
survdiff_lr_cv <- pairwise_survdiff(Surv(end, status) ~ landrace + trt, data = df_cv, 
    p.adjust.method = "bonferroni", rho = 0)
survdiff_lr_cv$p.value %>% kable() %>% kable_styling
```

|  | landrace=Chile de Agua, trt=0 | landrace=Chile de Agua, trt=20 | landrace=Taviche, trt=0 | landrace=Taviche, trt=20 | landrace=Tusta, trt=0 |
| --- | --- | --- | --- | --- | --- |
| landrace=Chile de Agua, trt=20 | 0.00e+00 | NA | NA | NA | NA |
| landrace=Taviche, trt=0 | 1.00e+00 | 0.0002527 | NA | NA | NA |
| landrace=Taviche, trt=20 | 5.59e-05 | 1.0000000 | 0.0162043 | NA | NA |
| landrace=Tusta, trt=0 | 1.00e+00 | 0.0000000 | 0.4258595 | 0 | NA |
| landrace=Tusta, trt=20 | 1.70e-06 | 1.0000000 | 0.0001637 | 1 | 0 |

```
# sink('central-valleys_landrace.txt'); print(survdiff_lr_cv$p.value);
# sink()


labels.wcoast <- c("Costeno Amarillo, 0% PEG", "Costeno Amarillo, 20% PEG", 
    "Costeno Rojo, 0% PEG", "Costeno Rojo, 20% PEG", "Piquin, 0% PEG", "Piquin, 20% PEG")
df_wcoast <- subset(df_0_20, region == "wcoast")
test.landrace.wcoast <- survfit(Surv(end, status) ~ landrace + trt, data = df_wcoast, 
    type = "kaplan-meier", conf.type = "log-log")
ggsurvplot(test.landrace.wcoast, data = df_wcoast, conf.int = T, pval = T, xlab = NULL, 
    legend = "right", ylab = "Not Germinated", surv.median.line = "hv", palette = "Paired", 
    title = "A) W Coast", legend.labs = labels.wcoast)
```

```
# ggsave(file = 'wcoast_landrace.jpg', dpi = 600)
ggsave(file = "Fig4A.tiff", dpi = 600)
```

```
## Saving 7 x 5 in image
```

```
survdiff_lr_wcoast <- pairwise_survdiff(Surv(end, status) ~ landrace + trt, 
    data = df_wcoast, p.adjust.method = "bonferroni", rho = 0)
survdiff_lr_wcoast$p.value %>% kable() %>% kable_styling
```

|  | landrace=Costeno Amarillo, trt=0 | landrace=Costeno Amarillo, trt=20 | landrace=Costeno Rojo, trt=0 | landrace=Costeno Rojo, trt=20 | landrace=Piquin, trt=0 |
| --- | --- | --- | --- | --- | --- |
| landrace=Costeno Amarillo, trt=20 | 1.0000000 | NA | NA | NA | NA |
| landrace=Costeno Rojo, trt=0 | 1.0000000 | 0.2014007 | NA | NA | NA |
| landrace=Costeno Rojo, trt=20 | 0.3071121 | 1.0000000 | 0.0000006 | NA | NA |
| landrace=Piquin, trt=0 | 0.0029968 | 1.0000000 | 0.0001138 | 0.4484091 | NA |
| landrace=Piquin, trt=20 | 0.0000000 | 0.0000000 | 0.0000000 | 0.0000000 | 0 |

```
# sink('wcoast_landrace.txt'); print(survdiff_lr_wcoast$p.value); sink()

labels.yucatan <- c("Dulce, 0% PEG", "Dulce, 20% PEG", "Paradito, 0% PEG", "Paradito, 20% PEG")
df_yucatan <- subset(df_0_20, region == "yucatan")
test.landrace.yucatan <- survfit(Surv(end, status) ~ landrace + trt, data = df_yucatan, 
    type = "kaplan-meier", conf.type = "log-log")
ggsurvplot(test.landrace.yucatan, data = df_yucatan, conf.int = T, pval = T, 
    xlab = NULL, legend = "right", ylab = "Not Germinated", surv.median.line = "hv", 
    palette = "Paired", title = "B) Yucatan", legend.labs = labels.yucatan)
```

```
# ggsave(file = 'yucatan_landrace.jpg', dpi = 600)
ggsave(file = "Fig4B.tiff", dpi = 600)
```

```
## Saving 7 x 5 in image
```

```
survdiff_lr_yucatan <- pairwise_survdiff(Surv(end, status) ~ landrace + trt, 
    data = df_yucatan, p.adjust.method = "bonferroni", rho = 0)
survdiff_lr_yucatan$p.value %>% kable() %>% kable_styling
```

|  | landrace=Dulce, trt=0 | landrace=Dulce, trt=20 | landrace=Paradito, trt=0 |
| --- | --- | --- | --- |
| landrace=Dulce, trt=20 | 6.1e-06 | NA | NA |
| landrace=Paradito, trt=0 | 0.0e+00 | 1 | NA |
| landrace=Paradito, trt=20 | 0.0e+00 | 0 | 0 |

```
# sink('yucatan_landrace.txt'); print(survdiff_lr_yucatan$p.value); sink()

labels.sm <- c("Tusta, 0% PEG", "Tusta, 20% PEG")
df_sm <- subset(df_0_20, region == "sierra-sur")
test.landrace.sm <- survfit(Surv(end, status) ~ landrace + trt, data = df_sm, 
    type = "kaplan-meier", conf.type = "log-log")
ggsurvplot(test.landrace.sm, data = df_sm, conf.int = T, pval = T, xlab = "Time (h)", 
    legend = "right", ylab = "Not Germinated", surv.median.line = "hv", title = "D) Sierra Sur", 
    legend.labs = labels.sm, palette = "Paired")
```

```
# ggsave(file = 'sierra-sur_landrace.jpg', dpi = 600)
ggsave(file = "Fig4D.tiff", dpi = 600)
```

```
## Saving 7 x 5 in image
```

```
survdiff_lr_sm <- pairwise_survdiff(Surv(end, status) ~ landrace + trt, data = df_sm, 
    p.adjust.method = "bonferroni", rho = 0)
survdiff_lr_sm$p.value %>% kable() %>% kable_styling
```

|  | landrace=Tusta, trt=0 |
| --- | --- |
| landrace=Tusta, trt=20 | 0 |

```
# sink('sierra-sur_landrace.txt'); print(survdiff_lr_sm$p.value); sink()
```

```
labels.cr <- c("E Coast, 0% PEG", "E Coast, 20% PEG", "W Coast, 0% PEG", "W Coast, 20% PEG")
df_costenorojo <- subset(df_0_20, landrace == "Costeno Rojo")
test.landrace.costenorojo <- survfit(Surv(end, status) ~ region + trt, data = df_costenorojo, 
    type = "kaplan-meier", conf.type = "log-log")
figsix <- list()
figsix[[1]] <- ggsurvplot(test.landrace.costenorojo, data = df_costenorojo, 
    conf.int = T, pval = T, xlab = "Time (h)", legend = "right", ylab = "Not Germinated", 
    surv.median.line = "hv", palette = "Paired", title = "A) Costeno Rojo", 
    legend.labs = labels.cr) + guides(col = guide_legend(ncol = 1))
# ggsave(file = 'costeno-rojo.jpg', dpi = 600) ggsave(file = 'Fig6A.tiff',
# dpi = 600)
survdiff_lr_costenorojo <- pairwise_survdiff(Surv(end, status) ~ region + trt, 
    data = df_costenorojo, p.adjust.method = "bonferroni", rho = 0)
survdiff_lr_costenorojo$p.value %>% kable() %>% kable_styling
```

|  | region=ecoast, trt=0 | region=ecoast, trt=20 | region=wcoast, trt=0 |
| --- | --- | --- | --- |
| region=ecoast, trt=20 | 1.3e-06 | NA | NA |
| region=wcoast, trt=0 | 6.0e-07 | 0 | NA |
| region=wcoast, trt=20 | 1.0e+00 | 0 | 2e-07 |

```
# sink('costeno-rojo.txt'); print(survdiff_lr_costenorojo$p.value); sink()


labels.tusta <- c("Central Valleys, 0% PEG", "Central Valleys, 20% PEG", "E Coast, 0% PEG", 
    "E Coast, 20% PEG", "Sierra Sur, 0% PEG", "Sierra Sur, 20% PEG")
df_tusta <- subset(df_0_20, landrace == "Tusta")
test.landrace.tusta <- survfit(Surv(end, status) ~ region + trt, data = df_tusta, 
    type = "kaplan-meier", conf.type = "log-log")
figsix[[2]] <- ggsurvplot(test.landrace.tusta, data = df_tusta, palette = "Paired", 
    conf.int = T, pval = T, xlab = "Time (h)", legend = "right", ylab = "Not Germinated", 
    surv.median.line = "hv", title = "B) Tusta", legend.labs = labels.tusta)
# ggsave(file = 'tusta.jpg', dpi = 600) ggsave(file = 'Fig6B.tiff', dpi =
# 600)
six <- arrange_ggsurvplots(figsix, print = T, ncol = 1, nrow = 2)
```

```
ggsave(file = "Fig6.tiff", six)
```

```
## Saving 7 x 5 in image
```

```
survdiff_lr_tusta <- pairwise_survdiff(Surv(end, status) ~ trt + region, data = df_tusta, 
    p.adjust.method = "bonferroni", rho = 0)
survdiff_lr_tusta$p.value %>% kable() %>% kable_styling
```

|  | trt=0, region=central-valleys | trt=0, region=ecoast | trt=0, region=sierra-sur | trt=20, region=central-valleys | trt=20, region=ecoast |
| --- | --- | --- | --- | --- | --- |
| trt=0, region=ecoast | 0.0000051 | NA | NA | NA | NA |
| trt=0, region=sierra-sur | 0.0015853 | 1 | NA | NA | NA |
| trt=20, region=central-valleys | 0.0000000 | 0 | 0 | NA | NA |
| trt=20, region=ecoast | 0.0000000 | 0 | 0 | 2.69e-05 | NA |
| trt=20, region=sierra-sur | 0.0000000 | 0 | 0 | 1.00e+00 | 8.72e-05 |

```
# sink('tusta.txt'); print(survdiff_lr_tusta$p.value); sink()
```

```
df_ecoast_cult <- subset(df_ecoast, cultivation %in% c("Backyard", "Forest", 
    "Milpa", "Plantation"))
labels.ecoast.cult <- c("Backyard, 0% PEG", "Backyard, 20% PEG", "Forest, 0% PEG", 
    "Forest, 20% PEG", "Milpa, 0% PEG", "Milpa, 20% PEG", "Plantation, 0% PEG", 
    "Plantation, 20% PEG")
test.ecoast.cult <- survfit(Surv(end, status) ~ cultivation + trt, data = df_ecoast_cult, 
    type = "kaplan-meier", na.action = na.omit)
ggsurvplot(test.ecoast.cult, data = df_ecoast_cult, conf.int = T, pval = T, 
    xlab = "Time (h)", legend = "right", ylab = "Not Germinated", surv.median.line = "hv", 
    palette = "Paired", legend.labs = labels.ecoast.cult) + guides(col = guide_legend(ncol = 1))
```

```
# ggsave(file = 'ecoast_cult.jpg', dpi = 600) ggsave(file =
# 'ecoast_cult.tiff', dpi = 600)
ggsave("Fig5.eps", dpi = 300, width = 2.5, units = "in")
```

```
## Saving 2.5 x 5 in image
```

```
## Warning in grid.Call.graphics(C_polygon, x$x, x$y, index): semi-
## transparency is not supported on this device: reported only once per page
```

```
survdiff_ecoast_cult <- pairwise_survdiff(Surv(end, status) ~ cultivation + 
    trt, data = df_ecoast_cult, p.adjust.method = "bonferroni", rho = 0)
survdiff_ecoast_cult$p.value %>% kable() %>% kable_styling
```

|  | cultivation=Backyard, trt=0 | cultivation=Backyard, trt=20 | cultivation=Forest, trt=0 | cultivation=Forest, trt=20 | cultivation=Milpa, trt=0 | cultivation=Milpa, trt=20 | cultivation=Plantation, trt=0 |
| --- | --- | --- | --- | --- | --- | --- | --- |
| cultivation=Backyard, trt=20 | 0.0000000 | NA | NA | NA | NA | NA | NA |
| cultivation=Forest, trt=0 | 0.0008204 | 0.0043862 | NA | NA | NA | NA | NA |
| cultivation=Forest, trt=20 | 0.0000000 | 0.0559641 | 7.1e-06 | NA | NA | NA | NA |
| cultivation=Milpa, trt=0 | 0.0000000 | 0.0000000 | 2.0e-07 | 0.0000000 | NA | NA | NA |
| cultivation=Milpa, trt=20 | 0.0000000 | 0.0383242 | 1.0e+00 | 0.0003785 | 0 | NA | NA |
| cultivation=Plantation, trt=0 | 0.0000000 | 0.0000000 | 7.2e-06 | 0.0000000 | 1 | 0.0000000 | NA |
| cultivation=Plantation, trt=20 | 0.0000000 | 0.0000000 | 1.0e+00 | 0.0000000 | 0 | 0.0006145 | 0 |

```
# sink('ecoast_cult.txt'); print(survdiff_ecoast_cult$p.value); sink()

df_ecoast_pop <- subset(df_ecoast, population.type %in% c("landrace", "letstand", 
    "wild"))
labels.ecoast.pop <- c("Landrace, 0% PEG", "Landrace, 20% PEG", "Letstand, 0% PEG", 
    "Letstand, 20% PEG", "Wild, 0% PEG", "Wild, 20% PEG")
test.ecoast.pop <- survfit(Surv(end, status) ~ population.type + trt, data = df_ecoast_pop, 
    type = "kaplan-meier", na.action = na.omit)
ggsurvplot(test.ecoast.pop, data = df_ecoast_pop, conf.int = T, pval = T, xlab = "Time (h)", 
    legend = "right", ylab = "Not Germinated", surv.median.line = "hv", title = "E Coast: Population types x [PEG]", 
    palette = "Paired", legend.labs = labels.ecoast.pop) + guides(col = guide_legend(ncol = 1))
```

```
# ggsave(file = 'ecoast_pop.jpg', dpi = 600) ggsave(file =
# 'ecoast_pop.tiff', dpi = 600)
survdiff_ecoast_pop <- pairwise_survdiff(Surv(end, status) ~ population.type + 
    trt, data = df_ecoast_pop, p.adjust.method = "bonferroni", rho = 0)
survdiff_ecoast_pop$p.value %>% kable() %>% kable_styling
```

|  | population.type=landrace, trt=0 | population.type=landrace, trt=20 | population.type=letstand, trt=0 | population.type=letstand, trt=20 | population.type=wild, trt=0 |
| --- | --- | --- | --- | --- | --- |
| population.type=landrace, trt=20 | 0.000000 | NA | NA | NA | NA |
| population.type=letstand, trt=0 | 0.000000 | 0.0000000 | NA | NA | NA |
| population.type=letstand, trt=20 | 0.000000 | 1.0000000 | 0 | NA | NA |
| population.type=wild, trt=0 | 0.000135 | 0.7836619 | 0 | 1.00e+00 | NA |
| population.type=wild, trt=20 | 0.000000 | 0.0007359 | 0 | 4.23e-05 | 3.8e-06 |

```
df_milpa <- subset(df_0_20, cultivation == "Milpa")
test.landrace.milpa <- survfit(Surv(end, status) ~ landrace + trt, data = df_milpa, 
    type = "kaplan-meier", conf.type = "log-log")
ggsurvplot(test.landrace.milpa, data = df_0_20, palette = "Paired", conf.int = T, 
    pval = T, xlab = "Time (h)", legend = "right", ylab = "Not Germinated", 
    title = "Milpa", surv.median.line = "hv")
```

```
# ggsave(file = 'milpa.jpg', dpi = 600) ggsave(file = 'milpa.tiff', dpi =
# 600)
ggsave("FigS4.eps", dpi = 300, width = 2.5, units = "in")
```

```
## Saving 2.5 x 5 in image
```

```
## Warning in grid.Call.graphics(C_polygon, x$x, x$y, index): semi-
## transparency is not supported on this device: reported only once per page
```

```
survdiff_milpa <- pairwise_survdiff(Surv(end, status) ~ landrace + trt, data = df_milpa, 
    p.adjust.method = "bonferroni", rho = 0)
survdiff_milpa$p.value %>% kable() %>% kable_styling
```

|  | landrace=Chile de Agua, trt=0 | landrace=Chile de Agua, trt=20 | landrace=Costeno Rojo, trt=0 | landrace=Costeno Rojo, trt=20 | landrace=Dulce, trt=0 | landrace=Dulce, trt=20 | landrace=Payaso, trt=0 | landrace=Payaso, trt=20 | landrace=Taviche, trt=0 | landrace=Taviche, trt=20 | landrace=Tusta, trt=0 |
| --- | --- | --- | --- | --- | --- | --- | --- | --- | --- | --- | --- |
| landrace=Chile de Agua, trt=20 | 0.0012916 | NA | NA | NA | NA | NA | NA | NA | NA | NA | NA |
| landrace=Costeno Rojo, trt=0 | 1.0000000 | 0.0000000 | NA | NA | NA | NA | NA | NA | NA | NA | NA |
| landrace=Costeno Rojo, trt=20 | 1.0000000 | 0.0000000 | 1.0000000 | NA | NA | NA | NA | NA | NA | NA | NA |
| landrace=Dulce, trt=0 | 0.0005402 | 0.0000000 | 0.4899686 | 0.0005440 | NA | NA | NA | NA | NA | NA | NA |
| landrace=Dulce, trt=20 | 1.0000000 | 1.0000000 | 0.0254234 | 0.1056892 | 0.0000669 | NA | NA | NA | NA | NA | NA |
| landrace=Payaso, trt=0 | 0.6766251 | 0.0000000 | 1.0000000 | 1.0000000 | 1.0000000 | 0.0003372 | NA | NA | NA | NA | NA |
| landrace=Payaso, trt=20 | 0.2619125 | 1.0000000 | 0.0001135 | 0.0001177 | 0.0000034 | 1.0000000 | 0.0000003 | NA | NA | NA | NA |
| landrace=Taviche, trt=0 | 1.0000000 | 0.0936014 | 0.2477376 | 1.0000000 | 0.0006878 | 1.0000000 | 0.0155881 | 0.6127277 | NA | NA | NA |
| landrace=Taviche, trt=20 | 0.0042433 | 1.0000000 | 0.0000000 | 0.0000000 | 0.0000000 | 1.0000000 | 0.0000000 | 1.0000000 | 0.0712988 | NA | NA |
| landrace=Tusta, trt=0 | 1.0000000 | 0.0000690 | 0.0008378 | 0.0119195 | 0.0000000 | 1.0000000 | 0.0000001 | 0.0106996 | 1.0000000 | 0.0000132 | NA |
| landrace=Tusta, trt=20 | 0.0000000 | 0.0000291 | 0.0000000 | 0.0000000 | 0.0000000 | 0.0000000 | 0.0000000 | 1.0000000 | 0.0000000 | 0.0015309 | 0 |

```
# sink('milpa.txt'); print(survdiff_milpa$p.value); sink()
```

```
df_0_20 %>% subset(population.type == "landrace") %>% distinct(landrace) %>% 
    kable() %>% kable_styling
```

| landrace |
| --- |
| Chile de Agua |
| Costeno Rojo |
| Costeno Amarillo |
| Dulce |
| Piquin |
| Tusta |
| Paradito |
| Guina Dahni |
| Chigole |
| Mareno |
| Solterito |
| Taviche |
| Frutescens |
| Chile Bolita |
| Mirasol |

```
df_0_20 %>% subset(population.type == "letstand") %>% distinct(landrace) %>% 
    kable() %>% kable_styling
```

| landrace |
| --- |
| Payaso |
| Frutescens |

```
df_0_20 %>% subset(population.type == "wild") %>% distinct(landrace) %>% kable() %>% 
    kable_styling
```

| landrace |
| --- |
| Chile de Monte |

```
df_0_20 %>% subset(cultivation == "Forest") %>% distinct(landrace) %>% kable() %>% 
    kable_styling
```

| landrace |
| --- |
| Chile de Monte |

```
df_0_20 %>% subset(cultivation == "Backyard") %>% distinct(landrace) %>% kable() %>% 
    kable_styling
```

| landrace |
| --- |
| Piquin |
| Paradito |
| Tusta |
| Chigole |
| Mareno |
| Solterito |
| Frutescens |
| Chile Bolita |
| Mirasol |

```
df_0_20 %>% subset(cultivation == "Milpa") %>% distinct(landrace) %>% kable() %>% 
    kable_styling
```

| landrace |
| --- |
| Chile de Agua |
| Dulce |
| Tusta |
| Costeno Rojo |
| Payaso |
| Taviche |

```
df_0_20 %>% subset(cultivation == "Plantation") %>% distinct(landrace) %>% kable() %>% 
    kable_styling
```

| landrace |
| --- |
| Costeno Rojo |
| Costeno Amarillo |
| Chile de Agua |
| Guina Dahni |

```
df_0_20 %>% subset(region == "central-valleys") %>% distinct(landrace) %>% kable() %>% 
    kable_styling
```

| landrace |
| --- |
| Chile de Agua |
| Tusta |
| Taviche |

```
df_0_20 %>% subset(region == "ecoast") %>% distinct(landrace) %>% kable() %>% 
    kable_styling
```

| landrace |
| --- |
| Tusta |
| Payaso |
| Guina Dahni |
| Costeno Rojo |
| Chigole |
| Mareno |
| Solterito |
| Chile de Monte |
| Frutescens |
| NA |
| Chile Bolita |
| Mirasol |

```
df_0_20 %>% subset(region == "sierra-sur") %>% distinct(landrace) %>% kable() %>% 
    kable_styling
```

| landrace |
| --- |
| Tusta |

```
df_0_20 %>% subset(region == "wcoast") %>% distinct(landrace) %>% kable() %>% 
    kable_styling
```

| landrace |
| --- |
| Costeno Rojo |
| Costeno Amarillo |
| Piquin |

```
df_0_20 %>% subset(region == "yucatan") %>% distinct(landrace) %>% kable() %>% 
    kable_styling
```

| landrace |
| --- |
| Dulce |
| Paradito |

```
df_ecoast_cult %>% subset(cultivation == "Forest") %>% distinct(landrace) %>% 
    kable() %>% kable_styling
```

| landrace |
| --- |
| Chile de Monte |

```
df_ecoast_cult %>% subset(cultivation == "Backyard") %>% distinct(landrace) %>% 
    kable() %>% kable_styling
```

| landrace |
| --- |
| Tusta |
| Chigole |
| Mareno |
| Solterito |
| Frutescens |
| Chile Bolita |
| Mirasol |

```
df_ecoast_cult %>% subset(cultivation == "Milpa") %>% distinct(landrace) %>% 
    kable() %>% kable_styling
```

| landrace |
| --- |
| Tusta |
| Payaso |

```
df_ecoast_cult %>% subset(cultivation == "Plantation") %>% distinct(landrace) %>% 
    kable() %>% kable_styling
```

| landrace |
| --- |
| Guina Dahni |
| Costeno Rojo |

```
sessionInfo()
```

```
## R version 3.5.3 (2019-03-11)
## Platform: x86_64-w64-mingw32/x64 (64-bit)
## Running under: Windows 10 x64 (build 19041)
## 
## Matrix products: default
## 
## locale:
## [1] LC_COLLATE=English_United States.1252 
## [2] LC_CTYPE=English_United States.1252   
## [3] LC_MONETARY=English_United States.1252
## [4] LC_NUMERIC=C                          
## [5] LC_TIME=English_United States.1252    
## 
## attached base packages:
## [1] stats     graphics  grDevices utils     datasets  methods   base     
## 
## other attached packages:
##  [1] DT_0.13            dplyr_0.8.3        kableExtra_1.1.0  
##  [4] knitr_1.23         RColorBrewer_1.1-2 survminer_0.4.5   
##  [7] ggpubr_0.2.2       magrittr_1.5       ggplot2_3.3.2     
## [10] mnormt_1.5-5       survival_2.44-1.1  pacman_0.5.1      
## 
## loaded via a namespace (and not attached):
##  [1] zoo_1.8-6         tidyselect_1.1.0  xfun_0.8         
##  [4] purrr_0.3.2       splines_3.5.3     lattice_0.20-38  
##  [7] colorspace_1.4-1  generics_0.0.2    vctrs_0.3.4      
## [10] viridisLite_0.3.0 htmltools_0.5.0   yaml_2.2.0       
## [13] survMisc_0.5.5    rlang_0.4.7       pillar_1.4.2     
## [16] glue_1.4.2        withr_2.1.2       lifecycle_0.1.0  
## [19] stringr_1.4.0     munsell_0.5.0     ggsignif_0.6.0   
## [22] gtable_0.3.0      rvest_0.3.4       htmlwidgets_1.5.1
## [25] evaluate_0.14     labeling_0.3      highr_0.8        
## [28] broom_0.5.2       Rcpp_1.0.2        xtable_1.8-4     
## [31] readr_1.3.1       scales_1.0.0      backports_1.1.4  
## [34] formatR_1.7       webshot_0.5.1     km.ci_0.5-2      
## [37] gridExtra_2.3     hms_0.5.0         digest_0.6.20    
## [40] stringi_1.4.3     KMsurv_0.1-5      grid_3.5.3       
## [43] tools_3.5.3       tibble_2.1.3      crayon_1.3.4     
## [46] tidyr_1.1.2       pkgconfig_2.0.2   Matrix_1.2-15    
## [49] xml2_1.2.1        data.table_1.12.2 httr_1.4.0       
## [52] rstudioapi_0.10   assertthat_0.2.1  rmarkdown_1.14   
## [55] R6_2.4.0          nlme_3.1-137      compiler_3.5.3
```
