# Supplementary material for: Germination response of diverse wild and landrace chile peppers (Capsicum spp.) under drought stress simulated with polyethylene glycol
Source: PLoS One. 2020 Nov 16;15(11):e0236001. doi: 10.1371/journal.pone.0236001 (PMC7668591; doi:10.1371/journal.pone.0236001)
Supplement: S3 File — P-values within each table were adjusted for multiple comparisons with a Bonferroni correction. (PDF) [file pone.0236001.s007.pdf]

S3 File

Table 1. Pairwise comparisons of Kaplan-Meier survival curve estimates presented in S2 Fig.

Level of PEG	0%	10%	15%
10%	0.485		
15%	**p<0.001**	**p<0.001**	
20%	**p<0.001**	**p<0.001**	**p<0.001**

Table 2. Ecozone × PEG: Pairwise comparisons of Kaplan-Meier survival curve estimates presented in Fig 2.

	0% PEG, Central Valleys	0% PEG, E Coast	0% PEG, Sierra Sur	0% PEG, W Coast	0% PEG, Yucatán	20% PEG, Central Valleys	20% PEG, E Coast	20% PEG, Sierra Sur	20% PEG, W Coast
0% PEG, E Coast	**p<0.001**								
0% PEG, Sierra Sur	1.000	1.000							
0% PEG, W Coast	**0.003**	**p<0.001**	**0.003**						
0% PEG, Yucatán	1.000	0.145	1.000	**0.0096**					
20% PEG, Central Valleys	**p<0.001**	**p<0.001**	**p<0.001**	**p<0.001**	**p<0.001**				
20% PEG, E Coast	**p<0.001**	**p<0.001**	**p<0.001**	**p<0.001**	**p<0.001**	**p<0.001**			
20% PEG, Sierra Sur	**p<0.001**	**p<0.001**	**p<0.001**	**p<0.001**	**p<0.001**	1.000	**p<0.001**		
20% PEG, W Coast	0.002	1.000	1.000	**p<0.001**	0.796	**p<0.001**	**p<0.001**	**p<0.001**	
20% PEG, Yucatán	**p<0.001**	**p<0.001**	**p<0.001**	**p<0.001**	**p<0.001**	**p<0.001**	1.000	**p<0.001**	**p<0.001**

Table 3. Cultivation × PEG: Pairwise comparisons of Kaplan-Meier survival curve estimates presented in Fig 3.

	Backyard, 0% PEG	Backyard, 20% PEG	Forest, 0% PEG	Forest, 20% PEG	Milpa, 0% PEG	Milpa, 20% PEG	Plantation, 0% PEG
Backyard, 20% PEG	**p<0.001**						
Forest, 0% PEG	**p<0.001**	0.008					
Forest, 20% PEG	**p<0.001**	0.027	**p<0.001**				
Milpa, 0% PEG	**p<0.001**	**p<0.001**	**p<0.001**	**p<0.001**			
Milpa, 20% PEG	**p<0.001**	**p<0.001**	1.00000	**p<0.001**	**p<0.001**		
Plantation, 0% PEG	**p<0.001**	**p<0.001**	**p<0.001**	**p<0.001**	1.000	**p<0.001**	
Plantation, 20% PEG	**p<0.001**	**p<0.001**	0.666	**p<0.001**	**p<0.001**	0.969	**p<0.001**

Table 4. Domestication × PEG: Pairwise comparisons of Kaplan-Meier survival curve estimates presented in S3 Fig.

	landrace, 0% PEG	landrace, 20% PEG	letstand, 0% PEG	letstand, 20% PEG	wild, 0% PEG
landrace, 20% PEG	**p<0.001**				
letstand, 0% PEG	**p<0.001**	**p<0.001**			
letstand, 20% PEG	**p<0.001**	1.000	**p<0.001**		
wild, 0% PEG	**p<0.001**	1.000	**p<0.001**	1.000	
wild, 20% PEG	**p<0.001**	**p<0.001**	**p<0.001**	**p<0.001**	**p<0.001**

Table 5. W Coast Landraces × PEG: Pairwise comparisons of Kaplan-Meier survival curve estimates presented in Fig 4A.

	Costeno Amarillo, 0% PEG	Costeno Amarillo, 20% PEG	Costeno Rojo, 0% PEG	Costeno Rojo, 20% PEG	Piquin, 0% PEG
Costeno Amarillo, 20% PEG	1.000				
Costeno Rojo, 0% PEG	1.000	0.201			
Costeno Rojo, 20% PEG	0.307	1.000	**p<0.001**		
Piquin, 0% PEG	**0.003**	1.000	**p<0.001**	0.448	
Piquin, 20% PEG	**p<0.001**	**p<0.001**	**p<0.001**	**p<0.001**	**p<0.001**

Table 6. Yucatán Landraces × PEG: Pairwise comparisons of Kaplan-Meier survival curve estimates presented in Fig 4B.

	Dulce, 0% PEG	Dulce, 20% PEG	Paradito, 0% PEG
Dulce, 20% PEG	**p<0.001**		
Paradito, 0% PEG	**p<0.001**	1.000	
Paradito, 20% PEG	**p<0.001**	**p<0.001**	**p<0.001**

Table 7. Central Valley Landraces × PEG: Pairwise comparisons of Kaplan-Meier survival curve estimates presented in Fig 4C.

	Chile de Agua, 0% PEG	Chile de Agua, 20% PEG	Taviche, 0% PEG	Taviche, 20% PEG	Tusta, 0% PEG
Chile de Agua, 20% PEG	**p<0.001**				
Taviche, 0% PEG	1.000	**p<0.001**			
Taviche, 20% PEG	**p<0.001**	1.000	**0.016**		
Tusta, 0% PEG	1.000	**p<0.001**	0.426	**p<0.001**	
Tusta, 20% PEG	**p<0.001**	1.000	**p<0.001**	1.000	**p<0.001**

Table 8. Sierra Sur Landrace × PEG: Pairwise comparisons of Kaplan-Meier survival curve estimates presented in Fig 4D.

	Tusta, 0% PEG
Tusta, 20% PEG	**p<0.001**

Table 9. E Coast Landraces × PEG: Pairwise comparisons of Kaplan-Meier survival curve estimates presented in Figs 5A and 5B.

	Chigole, 0% PEG	Chigole, 20% PEG	Chile Bolita, 0% PEG	Chile Bolita, 20% PEG	Chile de Monte, 0% PEG	Chile de Monte, 20% PEG	Costeno Rojo, 0% PEG	Costeno Rojo, 20% PEG	Frutescens, 0% PEG	Frutescens, 20% PEG	Guina Dahni, 0% PEG	Guina Dahni, 20% PEG	Mareno, 0% PEG	Mareno, 20% PEG	Mirasol, 0% PEG	Mirasol, 20% PEG	Payaso, 0% PEG	Payaso, 20% PEG	Solterito, 0% PEG	Solterito, 20% PEG	Tusta, 0% PEG
Chigole, 20% PEG	**p<0.001**																				
Chile Bolita, 0% PEG	1.000	**p<0.001**																			
Chile Bolita, 20% PEG	**p<0.001**	1.000	**p<0.001**																		
Chile de Monte, 0% PEG	**0.003**	1.000	**0.002**	0.433																	
Chile de Monte, 20% PEG	**p<0.001**	**0.007**	**p<0.001**	0.130	**p<0.001**																
Costeno Rojo, 0% PEG	1.000	**p<0.001**	1.000	**p<0.001**	**0.006**	**p<0.001**															
Costeno Rojo, 20% PEG	**0.001**	**0.022**	**p<0.001**	**p<0.001**	1.000	**p<0.001**	**p<0.001**														
Frutescens, 0% PEG	1.000	**p<0.001**	1.000	**p<0.001**	0.128	**p<0.001**	1.000	1.000													
Frutescens, 20% PEG	**p<0.001**	0.065	**p<0.001**	1.000	**0.002**	1.000	**p<0.001**	**p<0.001**	**p<0.001**												
Guina Dahni, 0% PEG	**p<0.001**	**p<0.001**	**p<0.001**	**p<0.001**	**p<0.001**	**p<0.001**	**p<0.001**	**p<0.001**	**p<0.001**	**p<0.001**											
Guina Dahni, 20% PEG	**p<0.001**	1.000	**p<0.001**	**p<0.001**	1.000	**p<0.001**	**p<0.001**	1.000	**0.021**	**p<0.001**	**p<0.001**										
Mareno, 0% PEG	1.000	**p<0.001**	1.000	**p<0.001**	**0.001**	**p<0.001**	1.000	**0.011**	0.288	**p<0.001**	**p<0.001**	**p<0.001**									
Mareno, 20% PEG	**p<0.001**	1.000	**p<0.001**	1.000	0.102	1.000	**p<0.001**	**0.004**	**p<0.001**	1.000	**p<0.001**	**0.005**	**p<0.001**								
Mirasol, 0% PEG	0.160	**p<0.001**	0.311	**p<0.001**	1.000	**p<0.001**	0.110	1.000	1.000	**p<0.001**	**p<0.001**	1.000	**0.004**	**p<0.001**							
Mirasol, 20% PEG	**p<0.001**	0.433	**p<0.001**	1.000	**0.007**	1.000	**p<0.001**	**p<0.001**	**p<0.001**	1.000	**p<0.001**	**p<0.001**	**p<0.001**	1.000	**p<0.001**						
Payaso, 0% PEG	**p<0.001**	**p<0.001**	**p<0.001**	**p<0.001**	**p<0.001**	**p<0.001**	**p<0.001**	**p<0.001**	**p<0.001**	**p<0.001**	0.817	**p<0.001**	0.355	**p<0.001**	**p<0.001**	**p<0.001**					
Payaso, 20% PEG	0.441	1.000	0.546	**0.015**	1.000	**p<0.001**	0.599	1.000	1.000	**p<0.001**	**p<0.001**	1.000	0.112	**0.003**	1.000	**p<0.001**	**p<0.001**				
Solterito, 0% PEG	0.097	**0.034**	0.070	**p<0.001**	1.000	**p<0.001**	0.149	1.000	1.000	**p<0.001**	**p<0.001**	1.000	**0.047**	**p<0.001**	1.000	**p<0.001**	**p<0.001**	1.000			
Solterito, 20% PEG	**p<0.001**	**0.005**	**p<0.001**	0.160	**p<0.001**	1.000	**p<0.001**	**p<0.001**	**p<0.001**	1.000	**p<0.001**	**p<0.001**	**p<0.001**	1.000	**p<0.001**	1.000	**p<0.001**	**p<0.001**	**p<0.001**		
Tusta, 0% PEG	1.000	**p<0.001**	1.000	**p<0.001**	**0.003**	**p<0.001**	1.000	**0.002**	1.000	**p<0.001**	**p<0.001**	**p<0.001**	1.000	**p<0.001**	**0.047**	**p<0.001**	**p<0.001**	0.398	0.060	**p<0.001**	
Tusta, 20% PEG	**p<0.001**	1.000	**p<0.001**	1.000	1.000	**0.033**	**p<0.001**	**p<0.001**	**p<0.001**	0.380	**p<0.001**	**0.018**	**p<0.001**	1.000	**p<0.001**	1.000	**p<0.001**	0.724	**p<0.001**	**0.022**	**p<0.001**

Table 10. E Coast Cultivation × PEG: Pairwise comparisons of Kaplan-Meier survival curve estimates presented in Fig 6.

	landrace, 0% PEG	landrace, 20% PEG	letstand, 0% PEG	letstand, 20% PEG	wild, 0% PEG
landrace, 20% PEG	**p<0.001**				
letstand, 0% PEG	**p<0.001**	**p<0.001**			
letstand, 20% PEG	**p<0.001**	1.000	**p<0.001**		
wild, 0% PEG	**p<0.001**	0.784	**p<0.001**	1.000	
wild, 20% PEG	**p<0.001**	**0.001**	**p<0.001**	**p<0.001**	**p<0.001**

Table 11. Costeno Rojo Ecozone × PEG: Pairwise comparisons of Kaplan-Meier survival curve estimates presented in Fig 7A.

	E Coast, 0% PEG	E Coast, 20% PEG	W Coast, 0% PEG
E Coast, 20% PEG	**p<0.001**		
W Coast, 0% PEG	**p<0.001**	**p<0.001**	
W Coast, 20% PEG	1.000	**p<0.001**	**p<0.001**

Table 12. Tusta Ecozone × PEG: Pairwise comparisons of Kaplan-Meier survival curve estimates presented in Fig 7B.

	Central Valleys, 0% PEG	E Coast, 0% PEG	Sierra Sur, 0% PEG	Central Valleys, 20% PEG	E Coast, 20% PEG
E Coast, 0% PEG	**p<0.001**				
Sierra Sur, 0% PEG	**0.002**	1.000			
Central Valleys, 20% PEG	**p<0.001**	**p<0.001**	**p<0.001**		
E Coast, 20% PEG	**p<0.001**	**p<0.001**	**p<0.001**	**p<0.001**	
Sierra Sur, 20% PEG	**p<0.001**	**p<0.001**	**p<0.001**	1.000	**p<0.001**
