# Supplementary material for: Germination response of diverse wild and landrace chile peppers (Capsicum spp.) under drought stress simulated with polyethylene glycol
Source: PLoS One. 2020 Nov 16;15(11):e0236001. doi: 10.1371/journal.pone.0236001 (PMC7668591; doi:10.1371/journal.pone.0236001)
Supplement: Attachment — Submitted filename: Response to Reviewers.docx [file pone.0236001.s008.docx]

Dr. Shahid Farooq

Academic Editor, PLOS ONE

25 September 2020

Dear Dr. Farooq,

Thank you for considering our article for publication in PLOS ONE. We are grateful to you and Dr. Gammoudi for the suggestions you provided. We feel that by addressing them we have improved the manuscript. We also have taken the opportunity to reread the manuscript and make limited edits for clarity’s sake.

Additionally, when addressing Dr. Gammoudi’s comments regarding the parameters for our heritability analysis we identified an error in its execution. As this analysis has no bearing on our objectives and was only mentioned once in the discussion, we have decided to remove it from the manuscript.

Below, we have responded to each issue addressed in the review. Our responses are marked with asterisks and are in plain type, while the comments are italicized.

We have made all changes to the manuscript in track changes, as suggested. We have returned a version of the revised manuscript that includes those track changes and one that has all changes accepted.

Data and analysis scripts underlying this manuscript have been archived on FigShare. A DOI has been created for the dataset: 10.6084/m9.figshare.12933854.

We look forward to hearing back from you.

Sincerely,

Dr. Kristin L. Mercer

Associate Professor

mercer.97@osu.edu

*PONE-D-20-19642
Germination response of diverse wild and landrace chile peppers (Capsicum spp.) under drought stress simulated with polyethylene glycol
PLOS ONE

Dear Dr. Mercer,

Thank you for submitting your manuscript to PLOS ONE. After careful consideration, we feel that it has merit but does not fully meet PLOS ONE’s publication criteria as it currently stands. Therefore, we invite you to submit a revised version of the manuscript that addresses the points raised during the review process.*

- *I have now received one review report. Based on the report and my opinion, the manuscript can be published in Plos One after necessary minor revisions.*
- *There are confusions on the seed germination parameters studied. Please clarify these.*
- *There are some typos and differences in the use of names of different accessions.*
- *Please make these corrections and submit a revised version for further evaluation.*

*Please submit your revised manuscript by Sep 26 2020 11:59PM. If you will need more time than this to complete your revisions, please reply to this message or contact the journal office at* [*plosone@plos.org*](mailto:plosone@plos.org)*. When you're ready to submit your revision, log on to* [*https://www.editorialmanager.com/pone/*](https://gcc02.safelinks.protection.outlook.com/?url=https%3A%2F%2Furldefense.com%2Fv3%2F__https%3A%2Fgcc02.safelinks.protection.outlook.com%2F%3Furl%3Dhttps*3A*2F*2Furldefense.com*2Fv3*2F__https*3A*2Fgcc02.safelinks.protection.outlook.com*2F*3Furl*3Dhttps*3A*2F*2Furldefense.com*2Fv3*2F__https*3A*2Fwww.editorialmanager.com*2Fpone*2F__*3B!!KGKeukY!hG501bXixFbpEkNxqkLA5x2AfqAiDF9SNiCrx39YkzPNmVq5YcAdasL1Xxijh7SfEg*24*26data*3D02*7C01*7C*7C47694f9271654b843cd808d842c40077*7Ced5b36e701ee4ebc867ee03cfa0d4697*7C0*7C0*7C637332756879361938*26sdata*3DiJHbuv1rZeNR1bUjrkocT*2F6NwgG0tw0TsORAgU7*2Bf8E*3D*26reserved*3D0__*3BJSUlJSUlJSUlJSUlJSUlJSUlJSUl!!KGKeukY!n518M49MQ-cFPPsTKyiIwRhNGU9QjyOQyEIsN2zihkN6-hJaBVm-rbH-JVp_yOb3CA*24%26data%3D02*7C01*7C*7Cef467fae1a8a48a5e79a08d84449642c*7Ced5b36e701ee4ebc867ee03cfa0d4697*7C0*7C1*7C637334429291542402%26sdata%3D0AAhTH7xspCDxVHMEOR1PRLpzzmwJ0jCdZePd5rGIiQ*3D%26reserved%3D0__%3BJSUlJSUlJSUlJSoqKioqKioqKioqJSUqKioqKioqJSUqKiolJSUlJSUlJSUlJSU!!KGKeukY!haohwiNsYBhwQWrabeKJIpSuPGAefXqhMA_f12cBzQ97waQhGjj2onKuVUTB2vdcKg%24&data=02%7C01%7C%7C3fdb8d0d6a5b42f0961608d8452646a1%7Ced5b36e701ee4ebc867ee03cfa0d4697%7C0%7C1%7C637335377984089873&sdata=z%2FP%2FJHuAiSiflpl5lDeX7LAtHjzJ62uN21EvinhMzvs%3D&reserved=0) *and select the 'Submissions Needing Revision' folder to locate your manuscript file.

We look forward to receiving your revised manuscript.

Kind regards,

Shahid Farooq, Ph.D.
Academic Editor
PLOS ONE

Journal Requirements:*

*When submitting your revision, we need you to address these additional requirements.*

*1. Please ensure that your manuscript meets PLOS ONE's style requirements, including those for file naming. The PLOS ONE style templates can be found at*

[*https://journals.plos.org/plosone/s/file?id=wjVg/PLOSOne_formatting_sample_main_body.pdf*](https://gcc02.safelinks.protection.outlook.com/?url=https%3A%2F%2Furldefense.com%2Fv3%2F__https%3A%2Fgcc02.safelinks.protection.outlook.com%2F%3Furl%3Dhttps*3A*2F*2Furldefense.com*2Fv3*2F__https*3A*2Fgcc02.safelinks.protection.outlook.com*2F*3Furl*3Dhttps*3A*2F*2Furldefense.com*2Fv3*2F__https*3A*2Fjournals.plos.org*2Fplosone*2Fs*2Ffile*3Fid*3DwjVg*PLOSOne_formatting_sample_main_body.pdf__*3BLw!!KGKeukY!hG501bXixFbpEkNxqkLA5x2AfqAiDF9SNiCrx39YkzPNmVq5YcAdasL1Xxi0UrBCfg*24*26data*3D02*7C01*7C*7C47694f9271654b843cd808d842c40077*7Ced5b36e701ee4ebc867ee03cfa0d4697*7C0*7C0*7C637332756879371898*26sdata*3D10GvSJhWbrzYRdvqFbmkrZtu3hdCZsoyjDfK0L4LyQc*3D*26reserved*3D0__*3BJSUlJSUlJSUlJSUlKiUlJSUlJSUlJSU!!KGKeukY!n518M49MQ-cFPPsTKyiIwRhNGU9QjyOQyEIsN2zihkN6-hJaBVm-rbH-JVoXgXUk-Q*24%26data%3D02*7C01*7C*7Cef467fae1a8a48a5e79a08d84449642c*7Ced5b36e701ee4ebc867ee03cfa0d4697*7C0*7C1*7C637334429291562304%26sdata%3DW0*2BRi9oFg7V*2FXE0tzVyKKMDH4vTeYdTSlI5bBSzJwOo*3D%26reserved%3D0__%3BJSUlJSUlJSUlJSoqKioqKioqKioqKioqKiUlKioqKioqKiUlKiUlJSUlJSUlJSUlJSUl!!KGKeukY!haohwiNsYBhwQWrabeKJIpSuPGAefXqhMA_f12cBzQ97waQhGjj2onKuVURalrnozw%24&data=02%7C01%7C%7C3fdb8d0d6a5b42f0961608d8452646a1%7Ced5b36e701ee4ebc867ee03cfa0d4697%7C0%7C1%7C637335377984099808&sdata=V4bfU6E10lwb2kCzwmGqUezWC7W032kDhBgq3%2Bc7Kwg%3D&reserved=0) *and*

[*https://journals.plos.org/plosone/s/file?id=ba62/PLOSOne_formatting_sample_title_authors_affiliations.pdf*](https://gcc02.safelinks.protection.outlook.com/?url=https%3A%2F%2Furldefense.com%2Fv3%2F__https%3A%2Fgcc02.safelinks.protection.outlook.com%2F%3Furl%3Dhttps*3A*2F*2Furldefense.com*2Fv3*2F__https*3A*2Fgcc02.safelinks.protection.outlook.com*2F*3Furl*3Dhttps*3A*2F*2Furldefense.com*2Fv3*2F__https*3A*2Fjournals.plos.org*2Fplosone*2Fs*2Ffile*3Fid*3Dba62*PLOSOne_formatting_sample_title_authors_affiliations.pdf__*3BLw!!KGKeukY!hG501bXixFbpEkNxqkLA5x2AfqAiDF9SNiCrx39YkzPNmVq5YcAdasL1XxhKy7vCbA*24*26data*3D02*7C01*7C*7C47694f9271654b843cd808d842c40077*7Ced5b36e701ee4ebc867ee03cfa0d4697*7C0*7C0*7C637332756879371898*26sdata*3DEcn062hsNO6TPWb1sWqFahLfDiPu8lgf4u3qsJsKkwg*3D*26reserved*3D0__*3BJSUlJSUlJSUlJSUlKiUlJSUlJSUlJSU!!KGKeukY!n518M49MQ-cFPPsTKyiIwRhNGU9QjyOQyEIsN2zihkN6-hJaBVm-rbH-JVr6Wy5yZg*24%26data%3D02*7C01*7C*7Cef467fae1a8a48a5e79a08d84449642c*7Ced5b36e701ee4ebc867ee03cfa0d4697*7C0*7C1*7C637334429291562304%26sdata%3DXDIknBJSMJu7c10jFmjpSDMK57zcMvrhDzmMVsex6PQ*3D%26reserved%3D0__%3BJSUlJSUlJSUlJSoqKioqKioqKioqKioqKiUlKioqKioqKiUlKiUlJSUlJSUlJSUlJQ!!KGKeukY!haohwiNsYBhwQWrabeKJIpSuPGAefXqhMA_f12cBzQ97waQhGjj2onKuVUQL9-Frnw%24&data=02%7C01%7C%7C3fdb8d0d6a5b42f0961608d8452646a1%7Ced5b36e701ee4ebc867ee03cfa0d4697%7C0%7C0%7C637335377984109765&sdata=hPGUTIH8GW59w40sZRvJTi1EX%2BlFkIs%2FYkLRuXE8u10%3D&reserved=0)

**We have reviewed PLOS ONE’s style requirements and ensured that our manuscript meets them.

*2. We note that you have stated that you will provide repository information for your data at acceptance. Should your manuscript be accepted for publication, we will hold it until you provide the relevant accession numbers or DOIs necessary to access your data. If you wish to make changes to your Data Availability statement, please describe these changes in your cover letter and we will update your Data Availability statement to reflect the information you provide.*

**Data and analysis scripts underlying this manuscript has been archived on FigShare. A DOI has been created for the dataset: https://doi.org/10.6084/m9.figshare.12933854

*3. Please include a caption for figure 5.*

**A caption has been added for figure 5 (manuscript lines 287-291).

*4. We note that Figure 1 in your submission contain map images which may be copyrighted. All PLOS content is published under the Creative Commons Attribution License (CC BY 4.0), which means that the manuscript, images, and Supporting Information files will be freely available online, and any third party is permitted to access, download, copy, distribute, and use these materials in any way, even commercially, with proper attribution. For these reasons, we cannot publish previously copyrighted maps or satellite images created using proprietary data, such as Google software (Google Maps, Street View, and Earth). For more information, see our copyright guidelines:* [*http://journals.plos.org/plosone/s/licenses-and-copyright*](https://gcc02.safelinks.protection.outlook.com/?url=https%3A%2F%2Furldefense.com%2Fv3%2F__https%3A%2Fgcc02.safelinks.protection.outlook.com%2F%3Furl%3Dhttps*3A*2F*2Furldefense.com*2Fv3*2F__https*3A*2Fgcc02.safelinks.protection.outlook.com*2F*3Furl*3Dhttps*3A*2F*2Furldefense.com*2Fv3*2F__http*3A*2Fjournals.plos.org*2Fplosone*2Fs*2Flicenses-and-copyright__*3B!!KGKeukY!hG501bXixFbpEkNxqkLA5x2AfqAiDF9SNiCrx39YkzPNmVq5YcAdasL1XxiCIoYPTA*24*26data*3D02*7C01*7C*7C47694f9271654b843cd808d842c40077*7Ced5b36e701ee4ebc867ee03cfa0d4697*7C0*7C0*7C637332756879381851*26sdata*3DzkTCGXf*2BPK1APsdUnIUVOkxlDPbxJwedrV6uthhRCkE*3D*26reserved*3D0__*3BJSUlJSUlJSUlJSUlJSUlJSUlJSUl!!KGKeukY!n518M49MQ-cFPPsTKyiIwRhNGU9QjyOQyEIsN2zihkN6-hJaBVm-rbH-JVqYAXJ9kA*24%26data%3D02*7C01*7C*7Cef467fae1a8a48a5e79a08d84449642c*7Ced5b36e701ee4ebc867ee03cfa0d4697*7C0*7C1*7C637334429291572264%26sdata%3DWJE4rB1KnFyWKJHyE3*2Ff2jfvSTnmMbdjwEdZuxBr1G0*3D%26reserved%3D0__%3BJSUlJSUlJSUlJSoqKioqKioqKioqKiUlKioqKioqKiUlKiolJSUlJSUlJSUlJSUl!!KGKeukY!haohwiNsYBhwQWrabeKJIpSuPGAefXqhMA_f12cBzQ97waQhGjj2onKuVUR_v9CJgg%24&data=02%7C01%7C%7C3fdb8d0d6a5b42f0961608d8452646a1%7Ced5b36e701ee4ebc867ee03cfa0d4697%7C0%7C1%7C637335377984119721&sdata=dbkEqhDlnvQK1lvPBjuTLnSjuVl%2BdAeQky8nLHuBbA4%3D&reserved=0)*.*

*We require you to either (1) present written permission from the copyright holder to publish these figures specifically under the CC BY 4.0 license, or (2) remove the figures from your submission:*

**The map in Figure 1 was created in the R statistical environment using a shape file for the in the public domain (available from naturalearthdata.com). We have included this information in the caption for Figure 1. On manuscript lines 133-138, it now reads as follows:

**Figure 1. The five biogeographic ecozones from which we collected chile pepper accessions in Mexico.** Identified on a principal component analysis (PCA) of elevation and bioclimatic factors (A), and by geographical distribution of accessions within those ecozones (B). This figure was created using data from WorldClim [42] and Natural Earth. The packages ‘FactoMineR’ [43] and ‘raster’ [44] were used in the R statistical environment (ver. 3.5; R Development Core Team, 2019).

*Reviewers' comments:

Reviewer's Responses to Questions*

***Comments to the Author*** *1. Is the manuscript technically sound, and do the data support the conclusions?

The manuscript must describe a technically sound piece of scientific research with data that supports the conclusions. Experiments must have been conducted rigorously, with appropriate controls, replication, and sample sizes. The conclusions must be drawn appropriately based on the data presented.*

*Reviewer #1: Yes*

*2. Has the statistical analysis been performed appropriately and rigorously?*

*Reviewer #1: Yes*

*3. Have the authors made all data underlying the findings in their manuscript fully available?

The* [*PLOS Data policy*](https://gcc02.safelinks.protection.outlook.com/?url=https%3A%2F%2Furldefense.com%2Fv3%2F__https%3A%2Fgcc02.safelinks.protection.outlook.com%2F%3Furl%3Dhttps*3A*2F*2Furldefense.com*2Fv3*2F__https*3A*2Fgcc02.safelinks.protection.outlook.com*2F*3Furl*3Dhttps*3A*2F*2Furldefense.com*2Fv3*2F__http*3A*2Fwww.plosone.org*2Fstatic*2Fpolicies.action*sharing__*3BIw!!KGKeukY!hG501bXixFbpEkNxqkLA5x2AfqAiDF9SNiCrx39YkzPNmVq5YcAdasL1XxhIKHOSBg*24*26data*3D02*7C01*7C*7C47694f9271654b843cd808d842c40077*7Ced5b36e701ee4ebc867ee03cfa0d4697*7C0*7C0*7C637332756879421676*26sdata*3DpdsbCGpylmHgtClaR0AAw4r6vv7hSo7sBcHx4MR06h0*3D*26reserved*3D0__*3BJSUlJSUlJSUlKiUlJSUlJSUlJSU!!KGKeukY!n518M49MQ-cFPPsTKyiIwRhNGU9QjyOQyEIsN2zihkN6-hJaBVm-rbH-JVoIcfiXsw*24%26data%3D02*7C01*7C*7Cef467fae1a8a48a5e79a08d84449642c*7Ced5b36e701ee4ebc867ee03cfa0d4697*7C0*7C0*7C637334429291632005%26sdata%3DGZFMrANq4cwEKFkS60ATLJ8E2Q6mjTD*2FR3eIs4lNveE*3D%26reserved%3D0__%3BJSUlJSUlJSUlJSoqKioqKioqKioqKiUlKioqKioqKiUlKiUlJSUlJSUlJSUlJSU!!KGKeukY!haohwiNsYBhwQWrabeKJIpSuPGAefXqhMA_f12cBzQ97waQhGjj2onKuVURYZvfoVw%24&data=02%7C01%7C%7C3fdb8d0d6a5b42f0961608d8452646a1%7Ced5b36e701ee4ebc867ee03cfa0d4697%7C0%7C0%7C637335377984179461&sdata=eumEQoaPRIEHb0FhE6lQmkHJOz8B0KHjKH5Owl7Sxe0%3D&reserved=0) *requires authors to make all data underlying the findings described in their manuscript fully available without restriction, with rare exception (please refer to the Data Availability Statement in the manuscript PDF file). The data should be provided as part of the manuscript or its supporting information, or deposited to a public repository. For example, in addition to summary statistics, the data points behind means, medians and variance measures should be available. If there are restrictions on publicly sharing data—e.g. participant privacy or use of data from a third party—those must be specified.*

*Reviewer #1: Yes*

*4. Is the manuscript presented in an intelligible fashion and written in standard English?

PLOS ONE does not copyedit accepted manuscripts, so the language in submitted articles must be clear, correct, and unambiguous. Any typographical or grammatical errors should be corrected at revision, so please note any specific errors here.*

*Reviewer #1: Yes*

*5. Review Comments to the Author

Please use the space provided to explain your answers to the questions above. You may also include additional comments for the author, including concerns about dual publication, research ethics, or publication ethics. (Please upload your review as an attachment if it exceeds 20,000 characters)*

*Reviewer #1: The work presented interesting data about pepper culture under stressful condition.

- Please avoid les colored area around the curve. In some figures, it is difficult to differentiate, colored lines are sufficient.*

**We appreciate the reviewer’s suggestion regarding the clarity of our survival curve figures. However, we feel it is important to include the shaded area around each line to indicate the scale of the statistical error associated with each curve. In most figures, we feel the shading for the 95% confidence intervals can be easily interpreted. However, we have removed the shaded areas from Figure 5 to improve clarity of the lines. Furthermore, we have added text to each figures caption explaining that the shaded areas are confidence intervals.

*- References must be added for the parameters.*

**Thank you for this suggestion to improve the manuscript. We have decided to remove the heritability analysis from the manuscript as it had little bearing on the objectives or discussion of the results. As such, the descriptions of these parameters has also been removed.

*- Line 288 : « while PEG significantly affected germination curves of most landraces in most regions by significantly slowing their descent and reducing the final proportion of ungerminated seeds »
Comment : It is rather ‘increasing the final proportion…..’, please verify.*

**Indeed, the addition of the PEG treatment increased the final proportion of ungerminated seeds in most cases. Thank you for this edit. It has been corrected in the manuscript (lines 271-275).

*- Line 306 : « While only one C. annuum accession was collected in the Sierra Sur (Fig 5A and Fig 5B;Table 8 in S3 File),… »
Comment : ….(Fig. 4D ; …..)*

**Thank you for bringing this error to our attention. We have corrected the sentence to refer to Fig 4D in line 292 of the manuscript.

*- Line 308, 322 : Please use the same name for the accession : Gui’ña dani or Guina Dahni or Guiña Dahni.*

**Thank you for this edit. We have corrected the formatting for this landrace name throughout the manuscript.

*- For median germination time, noted here : t50:
Comment: to note, there is a difference between the mean or median germination time, usually noted (MGT), and the time taken for cumulative germination to reach 50% of its maximum, usually noted (t50) (as it is described by Gammoudi et al. (2020), Efficiency of pepper seed invigoration through hydrogen peroxide priming to improve in vitro salt and drought stress tolerance).
In the current manuscript, it is neither this nor that, here, it is simply the time to reach the percentage 50%, so, for that it was noted : « none of the other seven accessions from the Eastern Coast—all backyard garden landraces—reached 50% germination (t50) under the 20% PEG treatment », whereas, in fact, for each curve, we have a MGT and t50. Please verify.*

**Thank you for identifying the ambiguity of our wording regarding t50/germination rate/median germination time. We have revised our analysis and the manuscript by removing the use and mention of germination rate/median germination time.

*6. PLOS authors have the option to publish the peer review history of their article (*[*what does this mean?*](https://gcc02.safelinks.protection.outlook.com/?url=https%3A%2F%2Furldefense.com%2Fv3%2F__https%3A%2Fgcc02.safelinks.protection.outlook.com%2F%3Furl%3Dhttps*3A*2F*2Furldefense.com*2Fv3*2F__https*3A*2Fgcc02.safelinks.protection.outlook.com*2F*3Furl*3Dhttps*3A*2F*2Furldefense.com*2Fv3*2F__https*3A*2Fjournals.plos.org*2Fplosone*2Fs*2Feditorial-and-peer-review-process*loc-peer-review-history__*3BIw!!KGKeukY!hG501bXixFbpEkNxqkLA5x2AfqAiDF9SNiCrx39YkzPNmVq5YcAdasL1XxjSZoNZsw*24*26data*3D02*7C01*7C*7C47694f9271654b843cd808d842c40077*7Ced5b36e701ee4ebc867ee03cfa0d4697*7C0*7C0*7C637332756879431633*26sdata*3DiiQFPB8*2BWTLpSWLb*2F9I8G9VaNaJzPUfVttbSYHMXR24*3D*26reserved*3D0__*3BJSUlJSUlJSUlJSolJSUlJSUlJSUlJSU!!KGKeukY!n518M49MQ-cFPPsTKyiIwRhNGU9QjyOQyEIsN2zihkN6-hJaBVm-rbH-JVrb-VFc_A*24%26data%3D02*7C01*7C*7Cef467fae1a8a48a5e79a08d84449642c*7Ced5b36e701ee4ebc867ee03cfa0d4697*7C0*7C0*7C637334429291632005%26sdata%3Dqslowj0Gvgf9qyBJFZlkb85KXBv3rcU8Wlc*2FXhDz14Y*3D%26reserved%3D0__%3BJSUlJSUlJSUlJSoqKioqKioqKioqKiolJSoqKioqKiolJSoqKiUlJSUlJSUlJSUlJSU!!KGKeukY!haohwiNsYBhwQWrabeKJIpSuPGAefXqhMA_f12cBzQ97waQhGjj2onKuVURdRi9I9A%24&data=02%7C01%7C%7C3fdb8d0d6a5b42f0961608d8452646a1%7Ced5b36e701ee4ebc867ee03cfa0d4697%7C0%7C0%7C637335377984189414&sdata=6wX3JyKAbRQ5X9jgcLskRUE%2Bx0FuNjdEow3MoRC00%2F4%3D&reserved=0)*). If published, this will include your full peer review and any attached files.

If you choose “no”, your identity will remain anonymous but your review may still be made public.*

***Do you want your identity to be public for this peer review?*** *For information about this choice, including consent withdrawal, please see our* [*Privacy Policy*](https://gcc02.safelinks.protection.outlook.com/?url=https%3A%2F%2Furldefense.com%2Fv3%2F__https%3A%2Fgcc02.safelinks.protection.outlook.com%2F%3Furl%3Dhttps*3A*2F*2Furldefense.com*2Fv3*2F__https*3A*2Fgcc02.safelinks.protection.outlook.com*2F*3Furl*3Dhttps*3A*2F*2Furldefense.com*2Fv3*2F__https*3A*2Fwww.plos.org*2Fprivacy-policy__*3B!!KGKeukY!hG501bXixFbpEkNxqkLA5x2AfqAiDF9SNiCrx39YkzPNmVq5YcAdasL1XxjIOmEujA*24*26data*3D02*7C01*7C*7C47694f9271654b843cd808d842c40077*7Ced5b36e701ee4ebc867ee03cfa0d4697*7C0*7C0*7C637332756879431633*26sdata*3DK9HcuuqUIGDKkEBuDp1ksd*2FtUGWiXx8tTYB*2F*2ByAx1hE*3D*26reserved*3D0__*3BJSUlJSUlJSUlJSUlJSUlJSUlJSUl!!KGKeukY!n518M49MQ-cFPPsTKyiIwRhNGU9QjyOQyEIsN2zihkN6-hJaBVm-rbH-JVqTbEXDKw*24%26data%3D02*7C01*7C*7Cef467fae1a8a48a5e79a08d84449642c*7Ced5b36e701ee4ebc867ee03cfa0d4697*7C0*7C0*7C637334429291641965%26sdata%3D5YC6W1025YgltbJ*2FDsCwwixLn0*2BTdtxo3D91FcD01aE*3D%26reserved%3D0__%3BJSUlJSUlJSUlJSoqKioqKioqKiolJSoqKioqKiolJSoqKiolJSUlJSUlJSUlJSUlJQ!!KGKeukY!haohwiNsYBhwQWrabeKJIpSuPGAefXqhMA_f12cBzQ97waQhGjj2onKuVUSt42VzaQ%24&data=02%7C01%7C%7C3fdb8d0d6a5b42f0961608d8452646a1%7Ced5b36e701ee4ebc867ee03cfa0d4697%7C0%7C0%7C637335377984199369&sdata=L%2BoGRadRErSC0kVF%2B4%2FmcysDhb5K9WJcgH5slxuygJM%3D&reserved=0)*.*

*Reviewer #1:* ***Yes:*** *Najet Gammoudi*
